# Security enhanced multi-factor biometric authentication scheme using bio-hash function

**DOI:** 10.1371/journal.pone.0176250

**Published:** 2017-05-01

**Authors:** Younsung Choi, Youngsook Lee, Jongho Moon, Dongho Won

**Affiliations:** 1 Department of Cyber Security, Howon University, Impi-Myeon, Gunsan-Si, Jeonrabuk-Do 573-718, Korea; 2 Department of Computer Engineering, Sungkyunkwan University, 2066 Seoburo, Suwon, Gyeonggido 440-746, Korea; King Saud University, SAUDI ARABIA

## Abstract

With the rapid development of personal information and wireless communication technology, user authentication schemes have been crucial to ensure that wireless communications are secure. As such, various authentication schemes with multi-factor authentication have been proposed to improve the security of electronic communications. Multi-factor authentication involves the use of passwords, smart cards, and various biometrics to provide users with the utmost privacy and data protection. Cao and Ge analyzed various authentication schemes and found that Younghwa An’s scheme was susceptible to a replay attack where an adversary masquerades as a legal server and a user masquerading attack where user anonymity is not provided, allowing an adversary to execute a password change process by intercepting the user’s ID during login. Cao and Ge improved upon Younghwa An’s scheme, but various security problems remained. This study demonstrates that Cao and Ge’s scheme is susceptible to a biometric recognition error, slow wrong password detection, off-line password attack, user impersonation attack, ID guessing attack, a DoS attack, and that their scheme cannot provide session key agreement. Then, to address all weaknesses identified in Cao and Ge’s scheme, this study proposes a security enhanced multi-factor biometric authentication scheme and provides a security analysis and formal analysis using Burrows-Abadi-Needham logic. Finally, the efficiency analysis reveals that the proposed scheme can protect against several possible types of attacks with only a slightly high computational cost.

## Introduction

Distributed, networked system’s allow users to efficiently access resources at their convenience. Web services such as on-line shopping and Internet banking have become common in today’s technological world, and this has given rise to serious demand for remote authentication processes that ensure transactions between users and servers are secure. In various server environments, user authentication schemes are required to implemented elevated levels of ownership. The first password-based scheme was introduced by Lamport in 1981, and since then, various studies have been carried out on the security, efficiency, and costs of authentication schemes. Existing remote authentication schemes are mainly implemented using a public key system, and in most cases, these can be divided into traditional certificate-based authentication schemes and identity-based authentication schemes according to the type of evidence they adopt for authentication. [1–9].

Various identity-based schemes have been proposed to provide secure, efficient, and practical authentication. One class is based on a pairing operation, which is practical but inefficient since a high computational cost is needed to carry out the pairing operation. The second is based on a particular hash function through which identity information is mapped to a point on an elliptic curve, resulting in a complicated structure. The third is a direct ID-based scheme that uses a general cryptographic hash function with a structure that is more simple than that of the second class. Due to this structure’s simplicity, authentication can be accomplished only through a three-way handshake. However, it is still easy for a malicious person to cary out an attack. When all of the problems of the three categories mentioned above are taken into account, secure direct identity-based authentication schemes provide the optimum design for mobile device users and real-time applications. [10–20].

Recently, identity-based authentication schemes with a hash function were further divided into three categories according to the methods used in the authentication procedure: (1) knowledge-based scheme, (2) object-based scheme, and (3) biometrics-based scheme. However, each type has its own outstanding performance and limitations [21–37]:

- knowledge-based authentication is simple, convenient, and efficient, but it is weak to information leaks to malicious persons due to the adoption of a password,
- object-based authentication, based on the physical possession of a device such as a smart card, allows an adversary to impersonate legitimate users in a situation where the smart card is lost,
- biometrics-based authentication shows better results than the two types described above. The biometric keys, such as fingerprints or facial features, cannot be lost and forgotten. However, biometric samples, such as facial images, can be captured in various system databases, so biometric keys can remain insecure.

Multi-factor biometric authentication combines the use of a password, biometrics, and smart card protection to improve security and prevent various types of attacks, and it is not affected by the aforementioned defects. Such schemes have recently become a focal point of research, mainly reflected in the work put forward by various researchers. In 2010, Li and Hwang proposed a novel scheme using identity and a public key system, and then Das extended the work of Li *et al*. and made improvements to their weak scheme in 2011. Younghwa An showed that Das’s proposed protocol failed to achieve mutual authentication for the server and user in 2012. However, Younghwa An allows for an adversary to masquerade as a legal server or as a user since mutual authentication is not provided. Cao and Ge attempted to improve on Younghwa An’s scheme, but their scheme also has various security problems. We show that Cao and Ge’s scheme is vulnerable to a biometric recognition error, slow wrong password detection, off-line password attack, user impersonation attack, ID guessing attack, a DoS attack, and also lacks session key agreement. This study then proposes a scheme to provide improved security by resolving the issues inherent to Cao and Ge’s scheme [38–44].

The remainder of this paper is organized as follows. Section 2 briefly introduces related work on the bio-hash function and smart card information to help better understand the details of this paper. Section 3 briefly introduces Cao and Ge’s scheme. Section 4 mainly discusses its weaknesses. Section 5 describes countermeasures to solve its problems. Section 6 details the countermeasures to protect against all attacks. Section 7 is devoted to a formal security analysis of the modified scheme by using Burrows-Abadi-Needham logic (BAN-logic), and it compares the results of a security analysis and efficiency analysis with the modified scheme and some existing authentication schemes. The results indicate that the modified scheme has a slightly high computational cost and can protect against several possible attacks. Section 8 then concludes this paper.

## Related works

In this section, the adversary’s capability, bio-hash function and information for a smart card are explained to have a better understanding of the content of this paper.

### Adversary’s capability

In this paper, we assume the following about a probabilistic, polynomial-time adversary to properly capture the security requirements of a multi-factor biometric authentication scheme that uses smart cards during the registration phase, password change phase, and login and authentication phase [45].

- The adversary is able to have complete control over all message exchanges between the protocol participants, including a user and a server. That is, the adversary can intercept, insert, modify, delete, and eavesdrop on messages exchanged among the two parties at will.
- The adversary can (1) extract sensitive information from the smart card of a user through a power analysis attack or (2) determine the user’s password, possibly via shoulder-surfing or by employing a malicious card reader. However, the adversary cannot compromise both the information of the smart card and the password of the user. It is otherwise clear that there is no way to prevent the adversary from impersonating the user if both factors have been compromised.

### Bio-hash function

A hash function refers to a one-way transformation function. The hash function takes an arbitrary input and returns a string with a fixed size, which is referred to as a hash value or as a message digest.

Due to the peculiarity and ability of biometrics to differentiate a particular person from others, various systems have adopted methods to solve authentication and verification problems. However, a small change in biometric data (a little information missing from the biometric, noise, or a change in the order of the data input) may result in a momentous change in the hash value due to the uncertainty inherent to the retrieval of biometric features. In other words, general hash functions result in large differences due to slight differences in input, and recognition errors easily result from slight biometric changes. To resolve this problem, a bio-function system is proposed and studied. In various studies on bio-hashing systems, the bio-hash function must adhere to the following properties:

- similar biometric information should have similar hash values,
- different biometric information should not have similar hashes,
- rotation and translation of the original template should not have a substantial impact on hash values,
- partial biometric information (with missing core and delta) should be matched if sufficient detailed matters are present.

The hash function’s certain class can be formulated to be everlasting to the order in which the input pattern is presented to the hash function, and such hash functions are known as bio-hash function or symmetric hash. So, the bio-hash function can resolve the recognition error of general hash function and can authenticate a legal user even if the user’s biometric information changes a little [46, 47].

### Smart card information

Various researchers have shown that physically monitoring the power consumption can extract confidential information stored in all smart cards, such as by using a simple power analysis and a differential power analysis. When a user forgets an own smart card, an adversary can analyze it and extract all information stored within. Variations of such schemes are weak to password acquisition attacks off-line where an adversary can be authenticated to the server without separately obtaining the user’s information for login and authentication, such as their ID, password and biometrics. Therefore, the security-enhanced authentication scheme needs to be studied even if all the information of a user’s smart card is revealed [48, 49].

## Review of Cao and Ge’s authentication scheme

The process for Cao and Ge’s authentication scheme is reviewed before conducting the security analysis. Their scheme includes three phases: registration phase, password change phase, and login and authentication phase. The server *S*_*i*_ stores a secret value *X*_*s*_ and a user account database, which includes the legal user’s authentication information [50]. For convenience, the notation used throughout this paper are summarized in Table 1.

**Table 1:** Notation.

Notation	Description	Notation	Description
*U*_*i*_	User	*B*_*i*_	*U*_*i*_’s biometric template
*S*_*i*_	Server	*h*(⋅)	General hash function
*ID*_*i*_	User’s identity	*H*(⋅)	Bio-hash function
*PW*_*i*_	User’s password	*n*_*i*_	Counter number
*R*_*c*_	A random number generated by *U*_*i*_	⊕	Bitwise XOR operation
*R*_*s*_	A random number generated by *S*_*i*_	‖	Concatenation operation
*X*_*s*_	Secret key generated by *S*_*i*_	*T*_*i*_	*i*th timestamp

### Registration phase

**Fig 1:**
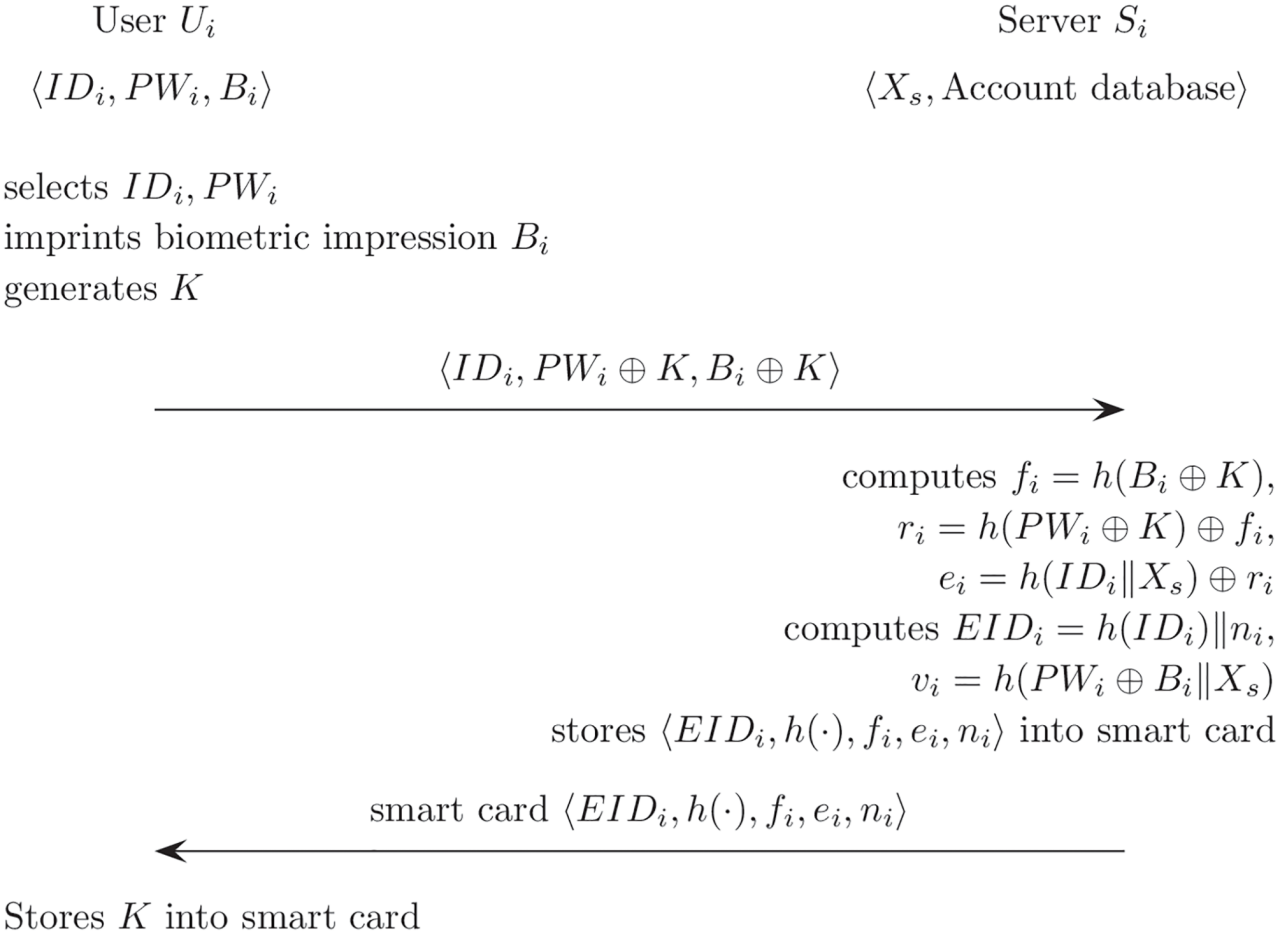
Registration phase for Cao and Ge’s authentication scheme.

This phase is the first to be performed once the *U*_*i*_ registers itself with the server *S*_*i*_. Fig 1 describes the registration phase for Cao and Ge’s scheme.

(R1) *U*_*i*_ selects *ID*_*i*_, *PW*_*i*_ and imprints its own *B*_*i*_, and generates *K*. Then, *U*_*i*_ sends the identity *ID*_*i*_, password information (*PW*_*i*_ ⊕ *K*), and biometric information (*B*_*i*_ ⊕ *K*) to the server *S*_*i*_ by using a secure channel.
(R2) *S*_*i*_ computes *f*_*i*_ = *h*(*B*_*i*_ ⊕ *K*), *r*_*i*_ = *h*(*PW*_*i*_ ⊕ *K*) ⊕ *f*_*i*_, and *e*_*i*_ = *h*(*ID*_*i*_‖*X*_*s*_) ⊕ *r*_*i*_.
(R3) *S*_*i*_ creates an entry for user *ID*_*i*_ and stores *n*_*i*_ on this entry in database. Then, *S*_*i*_ computes *EID*_*i*_ = *h*(*ID*_*i*_)‖*n*_*i*_ and stores *EID*_*i*_ to the entry.
(R4) *S*_*i*_ computes *v*_*i*_ = *h*(*PW*_*i*_ ⊕ *B*_*i*_‖*X*_*s*_).
(R5) *S*_*i*_ sends a smart card to *U*_*i*_. It contains 〈*EID*_*i*_, *h*(⋅), *f*_*i*_, *e*_*i*_, *n*_*i*_〉 using a secure channel. Then *U*_*i*_ stores *K* in the smart card.

### Password change phase

**Fig 2:**
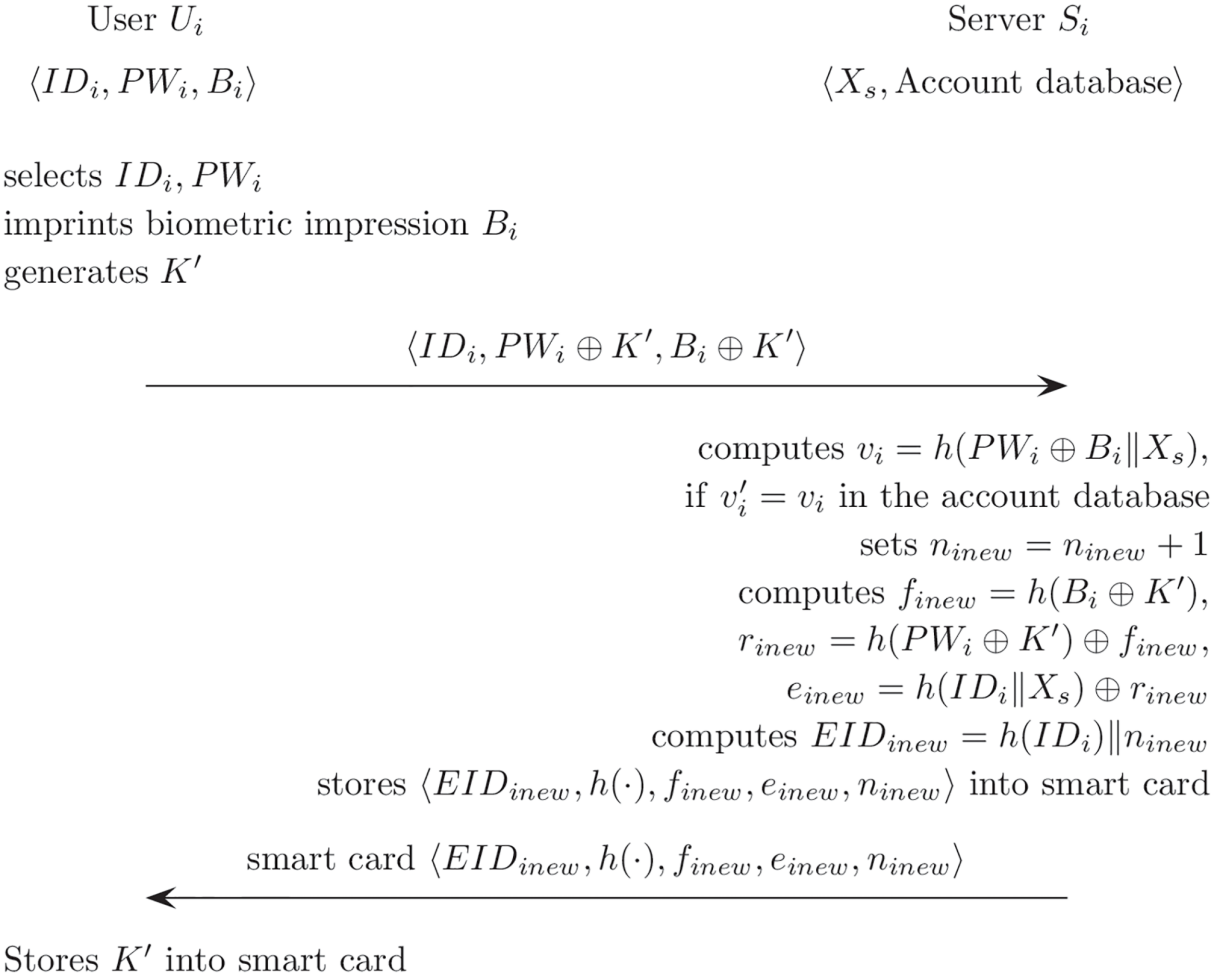
Password change phase on Cao and Ge’s authentication scheme.

The password change phase is carried out when *U*_*i*_ wants to change the password or the smart card is lost. Fig 2 describes the password change phase on Cao and Ge’s scheme.

(RR1) *U*_*i*_ submits the *ID*_*i*_ to *S*_*i*_, password information (*PW*_*i*_ ⊕ *K*′), and biometric information (*B*_*i*_ ⊕ *K*′) via a secure channel, *K*′ is the new random number.
(RR2) *S*_*i*_ computes $v_{i}^{\prime }=h\left(h\left(PW_{i}\right)⊕h\left(B_{i}\right)⊕X_{s}\right)$ and compares $v_{i}^{\prime }$ with *v*_*i*_ in the account database. If they are not the same, this phase is terminated.
(RR3) Otherwise, *S*_*i*_ computes *n*_*inew*_ = *n*_*i*_+1. Then, *S*_*i*_ performs the following computations; *f*_*inew*_ = *h*(*B*_*i*_ ⊕ *K*′), *r*_*inew*_ = *h*(*PW*_*i*_ ⊕ *K*′) ⊕ *f*_*inew*_, *e*_*inew*_ = *h*(*ID*_*i*_ ⊕ *X*_*s*_) ⊕ *r*_*inew*_.
(RR4) *S*_*i*_ sends *U*_*i*_ a new smart card that contains 〈*EID*_*i*_, *h*(⋅), *f*_*inew*_, *e*_*inew*_, *n*_*inew*_〉 by using secure channel. Then *U*_*i*_ stores the random number *K*′ in the smart card.

### Login and authentication phase

**Fig 3:**
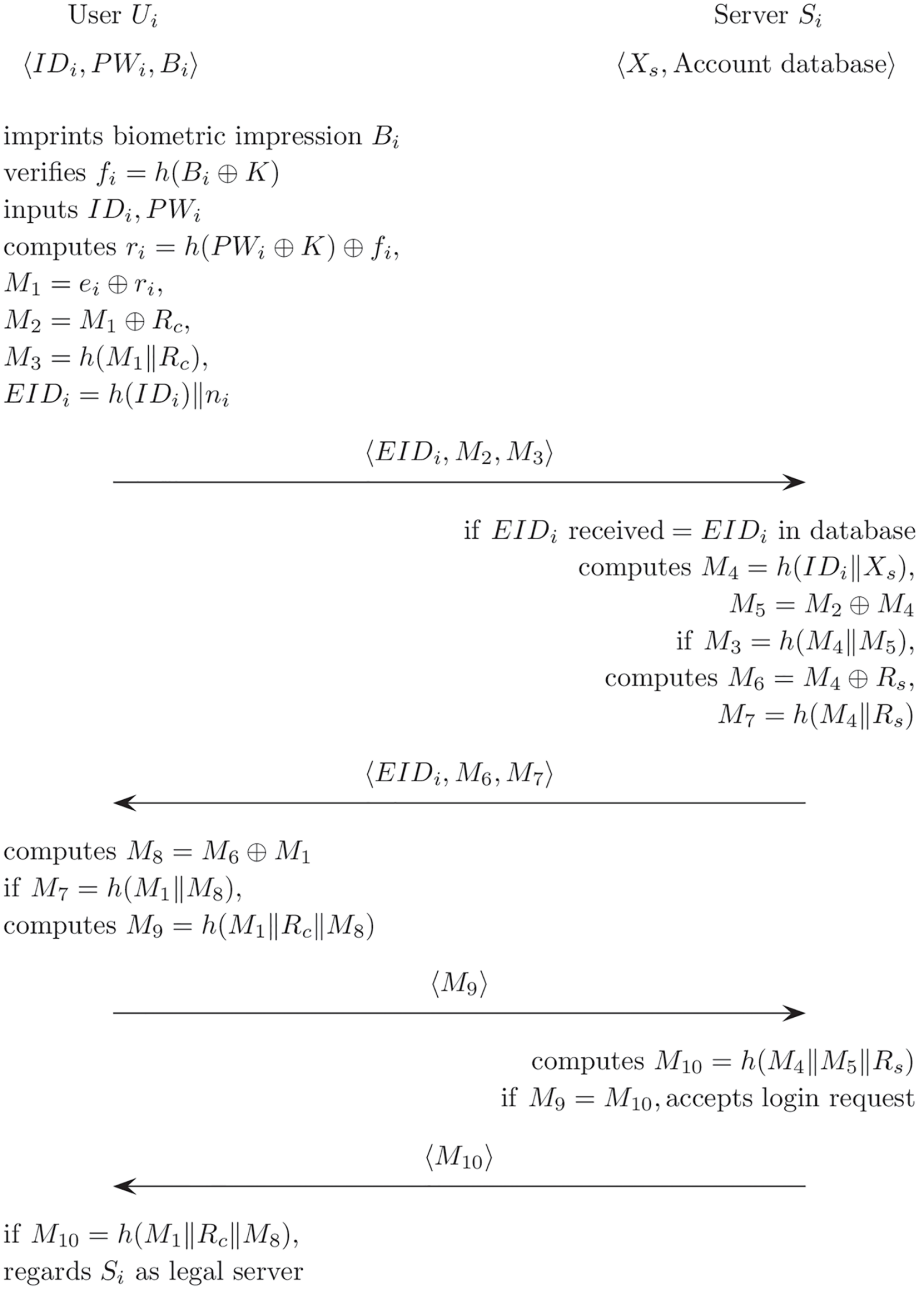
Login and authentication phase for Cao and Ge’s authentication scheme.

*U*_*i*_ executes the following steps when *U*_*i*_ wants to authenticate remote *S*_*i*_. Fig 3 describes the login and authentication phase on Cao and Ge’s scheme.

(L1) *U*_*i*_ imprints *B*_*i*_ using a biological feature extraction device, and it computes the information *h*(*B*_*i*_ ⊕ *K*) using *K* stored in the smart card. *U*_*i*_ can proceed only if *h*(*B*_*i*_ ⊕ *K*) matches *f*_*i*_.
(L2) *U*_*i*_ inputs the *ID*_*i*_ and *PW*_*i*_ and then, the smart card computes
  $$\begin{array}{ccc} r_{i} & = & h\left(PW_{i}⊕K\right)⊕f_{i},\\ M_{1} & = & e_{i}⊕r_{i},M_{2}=M_{1}⊕R_{c}\\ M_{3} & = & h\left(M_{1}∥R_{c}\right),EID_{i}=h\left(ID_{i}\right)∥n_{i}. \end{array}$$
(L3) The login request message 〈*EID*_*i*_, *M*_2_, *M*_3_〉 is then sent from *U*_*i*_ to *S*_*i*_.

The server *S*_*i*_ executes the authentication phase when the message is received.

(A1) *S*_*i*_ makes sure that *EID*_*i*_ satisfies the original format using the database entry and checks the *ID*_*i*_ for the authentication phase.
(A2) If the *ID*_*i*_ is valid when compared with database of *S*_*i*_, *S*_*i*_ computes
  $$\begin{array}{c} M_{4}=h\left(ID_{i}∥X_{s}\right),M_{5}=M_{2}⊕M_{4}. \end{array}$$
(A3) If *M*_3_ is the same as *h*(*M*_4_‖*M*_5_), *S*_*i*_ computes
  $$\begin{array}{ccc} M_{6} & = & M_{4}⊕R_{s},\\ M_{7} & = & h(M_{4}∥R_{s}). \end{array}$$
  Then, *S*_*i*_ sends the message 〈*M*_6_, *M*_7_〉 to *U*_*i*_.
(A4) *U*_*i*_ computes *M*_8_ and verifies whether *M*_7_ = *h*(*M*_1_‖*M*_8_) or not. If they are equal, *U*_*i*_ calculates *M*_9_.
  $$\begin{array}{ccc} M_{8} & = & M_{6}⊕M_{1},\\ M_{9} & = & h(M_{1}∥R_{c}∥M_{8}). \end{array}$$
(A5) *U*_*i*_ sends the message 〈*M*_9_〉 to *S*_*i*_.
(A6) After receiving 〈*M*_9_〉, *S*_*i*_ makes sure that *M*_9_ is equal to *M*_10_ = *h*(*M*_4_‖*M*_5_‖*R*_*s*_) and then accepts the user’s login request. *S*_*i*_ sends *M*_10_ to *U*_*i*_.
  $$\begin{array}{c} M_{10}=h(M_{4}∥M_{5}∥R_{s}) \end{array}$$
(A7) Upon receiving 〈*M*_10_〉, *U*_*i*_ makes sure that *M*_10_ is equal to *h*(*M*_1_‖*R*_*c*_‖*M*_8_) and then regards *S*_*i*_ as a legal server.
  $$\begin{array}{c} M_{10}=h(M_{1}∥R_{c}∥M_{8}) \end{array}$$

## Cryptanalysis of Cao and Ge’s authentication scheme

We analyze Cao and Ge’s authentication scheme and identify various security vulnerabilities, including a biometric recognition error, slow wrong password detection, off-line password attack, user impersonation attack, ID guessing attack, DoS attack, and a lack of session key agreement.

### Biometric recognition error

**Fig 4:**
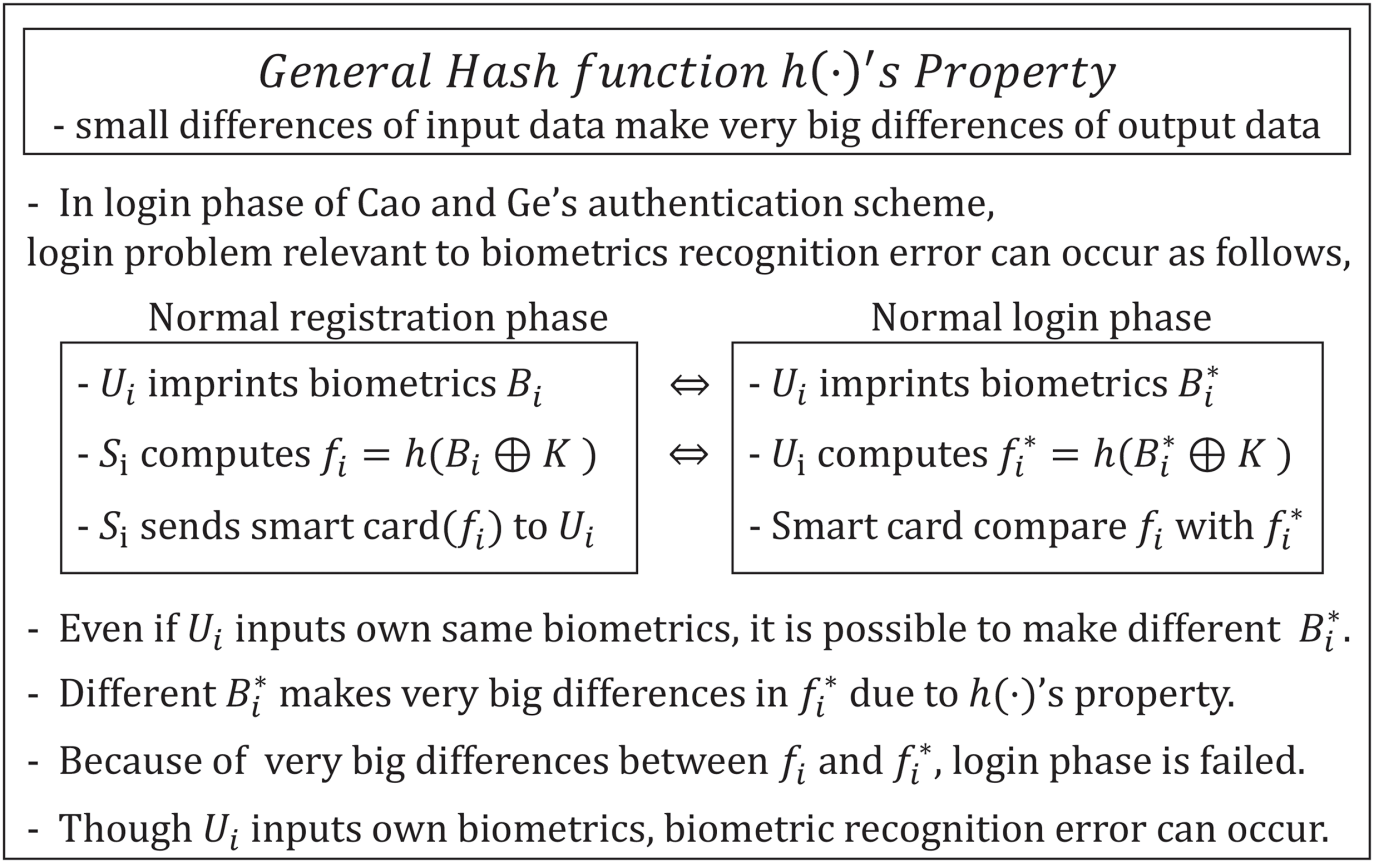
Biometric recognition error on Cao and Ge’s authentication scheme.

Cao and Ge’s authentication scheme only uses a general hash function to provide checking biometrics. However, the hash function has a property that causes a slight difference in the input data to result in a very large difference in the output data. Fig 4 describes the biometric recognition error in Cao and Ge’s scheme. The output of the imprinted biometrics is not always constant, so biometrics generally have instances of false acceptance and false rejection. Therefore, even when *U*_*i*_ imprints biometrics in the device, it is possible to output a different $B_{i}^{*}$. Therefore, the same user can generate a different output, such as that with *B*_*i*_ during the registration phase and $B_{i}^{*}$ during the login phase. The differences between *B*_*i*_ and $B_{i}^{*}$ can result in big differences in *f*_*i*_ and $f_{i}^{*}$, and this difference between *f*_*i*_ and $f_{i}^{*}$ results in a biometric recognition error in the login phase. Therefore, a normal user does not pass the user biometric verification stage because the smart card compares the computed $f_{i}^{*}$ to *f*_*i*_, which is stored within the smart card. Therefore, even though *U*_*i*_ imprints his/her own biometrics, a biometric recognition error can occur. Thus, the smart card needs to be implemented using more advanced techniques, such as a bio-hash function, to improve the biometrics verification process [51].

### Slow wrong password detection

**Fig 5:**
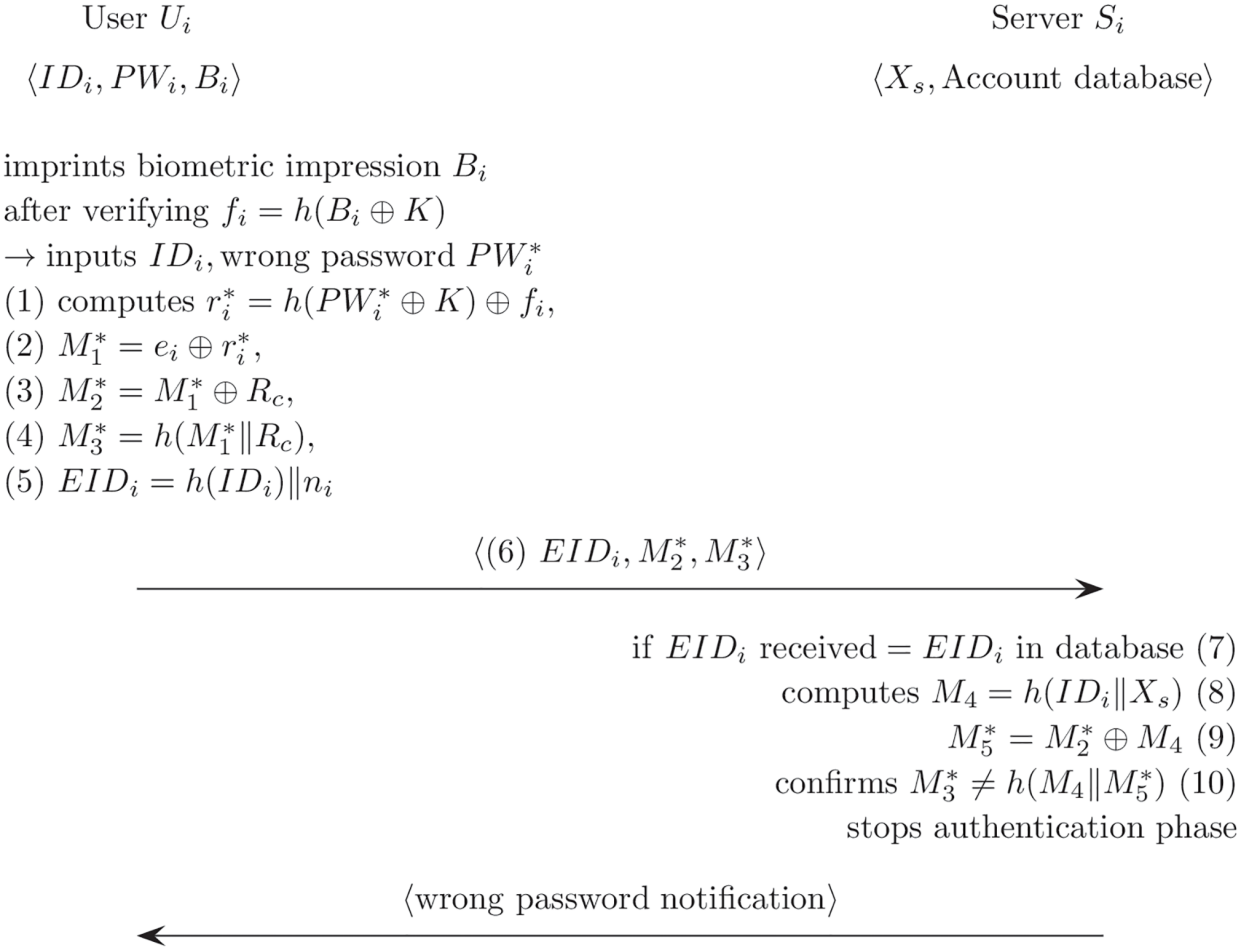
Slow wrong password detection on Cao and Ge’s authentication scheme.

Slow wrong password detection refers to instances in which the user cannot know of a mistake immediately when inputing the wrong password, and the user can know when server *S*_*i*_ notifies there is a wrong user password. In Cao and Ge’s authentication scheme, the user’s smart card cannot verify the accuracy of the user password during the login phase. Only *S*_*i*_ verifies a legal user by comparing the similarities between *M*_3_ and *h*(*M*_4_‖*M*_5_) during authentication phase. Fig 5 specifically describes how slowly the wrong password is detected in Cao and Ge’s scheme. Concretely, *U*_*i*_ inputs *ID*_*i*_ and *PW*_*i*_ after the biometric verification, then if *U*_*i*_ selects a wrong password $PW_{i}^{*}$, the smart card is unaware that the password is incorrect. The smart card does not check the $PW_{i}^{*}$, and it only computes various values $\langle r_{i}^{*},M_{1}^{*},M_{2}^{*},M_{3}^{*},EID_{i}\rangle$ using $PW_{i}^{*}$ for login and authentication. The smart card then sends $\langle EID_{i},M_{2}^{*},M_{3}^{*}\rangle$.

*S*_*i*_ is unable to immediately confirm the wrong password after receiving the messages $\langle EID_{i},M_{2}^{*},M_{3}^{*}\rangle$. First, *S*_*i*_ verifies the received *EID*_*i*_ using *EID*_*i*_ in the database, and then computes *M*_4_ = *h*(*ID*_*i*_‖*X*_*s*_) and $M_{5}^{*}=M_{2}^{*}⊕M_{4}$. Then, because $M_{3}^{*}$ is same as $h(M_{4}∥M_{5}^{*})$, *S*_*i*_ eventually confirms that the received messages are not normal, and maybe *U*_*i*_ could have input the wrong password. Basically, *S*_*i*_ sends the wrong password notification to *U*_*i*_. In detail, Cao and Ge’s scheme requires a lengthy phase that includes value computation and message transmission before confirming that the user input the wrong password. Therefore, a smart card is needed to provide a fast wrong password detection technique during login. When *U*_*i*_ inputs the wrong password during the login phase, the smart card needs to quickly identify the incorrect password and should immediately notify *U*_*i*_ of the mistake.

### Off-line password attack

**Fig 6:**
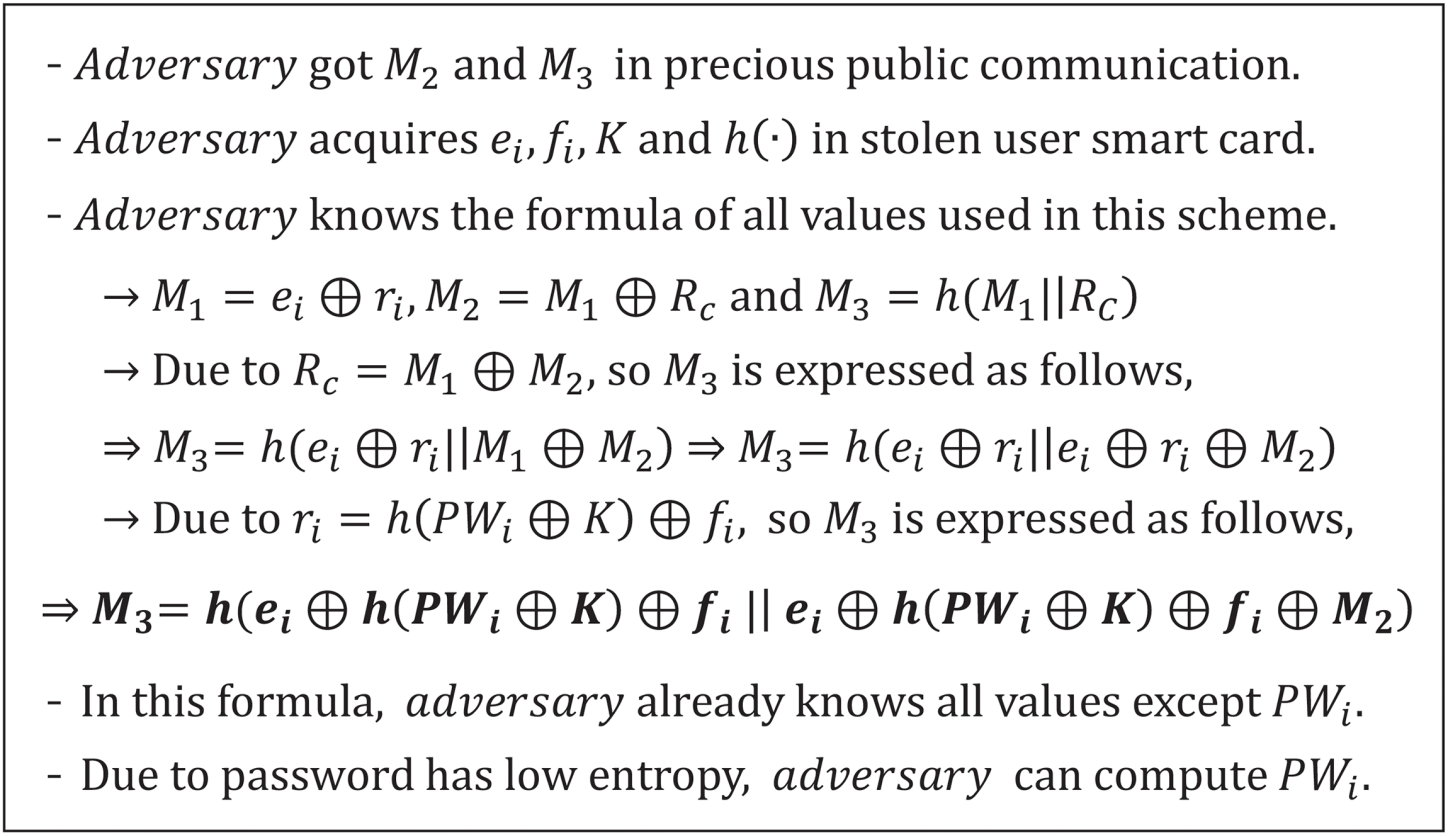
Off-line password attack on Cao and Ge’s authentication scheme.

In Cao and Ge’s scheme, an adversary can compute the user’s password by using public messages and the user’s smart card, obtaining *M*_2_ and *M*_3_ from public messages between the user and the server. Fig 6 provides a detailed description of the off-line password attack for Cao and Ge’s scheme. Kocher *et al*. and Messerges *et al*. claim that the all confidential information that is generally stored in smart cards could be extracted through various forms, such as monitoring the power consumption. Therefore, if a user loses a smart card, all of the information in the smart card can be revealed by an adversary. The smart card stores various types of information, including user login and authentication, so the adversary can acquire the *e*_*i*_, *f*_*i*_, *K*, and hash function *h*(⋅) values from the user’s smart card. The adversary knows the formula for all values used in Cao and Ge’s scheme as follows:

$$\begin{array}{ccc} M_{1}=e_{i}⊕r_{i} & , & M_{2}=M_{1}⊕R_{c}\\ M_{3}=h\left(M_{1}∥R_{c}\right) & , & r_{i}=h\left(PW_{i}⊕K\right)⊕f_{i}. \end{array}$$

The adversary uses the determined values, messages, and formula to compute the *M*_3_ formula, as follows:

$$\begin{array}{ccc} M_{3} & = & h(e_{i}⊕h\left(PW_{i}⊕f_{i}\right)⊕f_{i}∥e_{i}⊕h\left(PW_{i}⊕K\right)⊕f_{i}⊕M_{2}). \end{array}$$

The adversary then knows all values in this formula, except for *PW*_*i*_. Therefore, the adversary can easily determine the user’s password *PW*_*i*_ by mounting an off-line password guessing attack because the password *PW*_*i*_ is not long enough and has a low level of entropy. If the adversary knows the *PW*_*i*_, various attacks can be facilitated by using the user’s password. Therefore, the password needs to be protected by using other values that are not stored in the smart the card with a high entropy, such as biometric information [52].

### User impersonation attack

**Fig 7:**
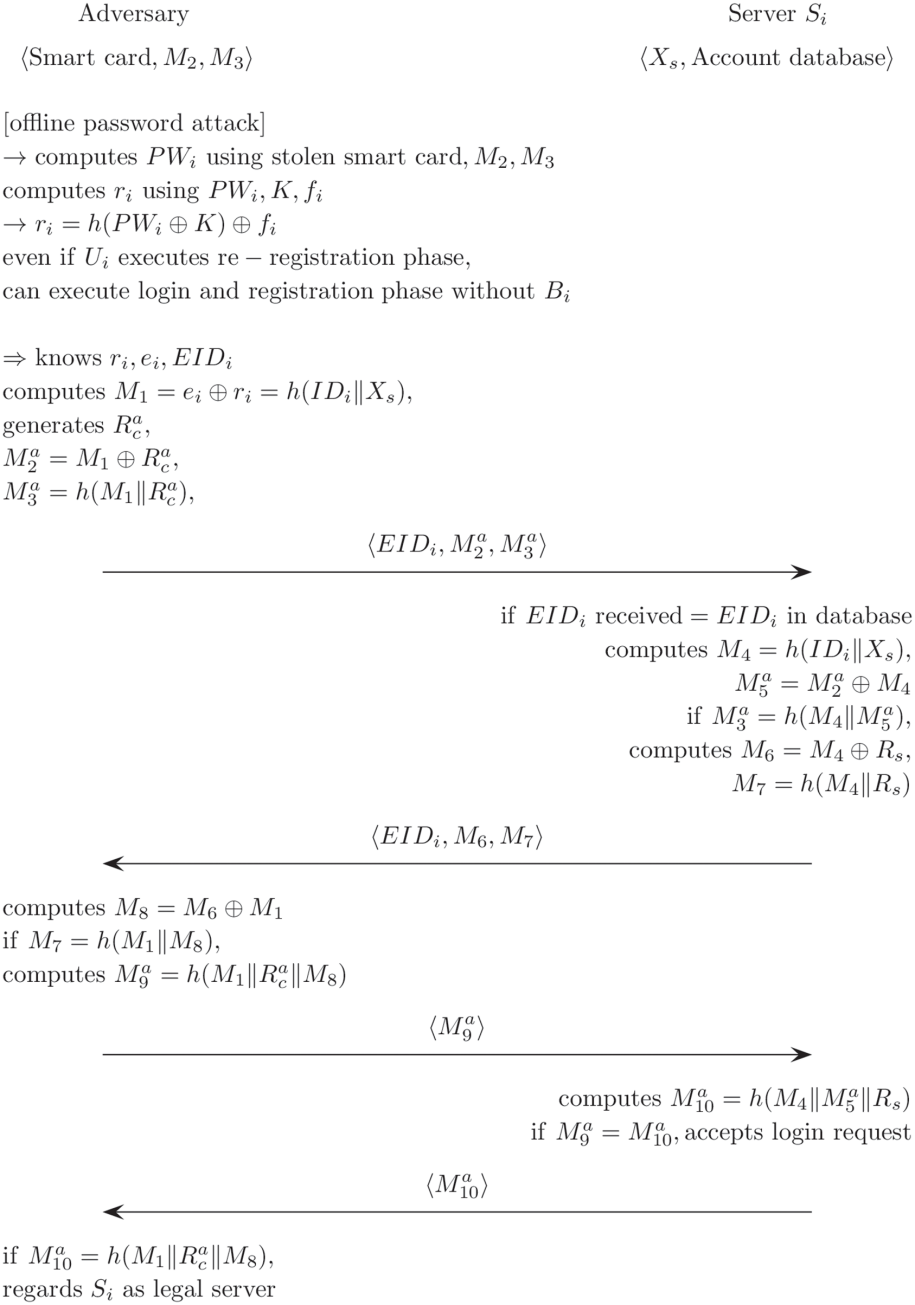
User impersonation attack on Cao and Ge’s authentication scheme.

In Cao and Ge’s scheme, an adversary can be authenticated with the server by using the user’s smart card and the password without access to the user’s biometric information. Fig 7 describes in detail a user impersonation attack for Cao and Ge’s authentication scheme. In further detail, when an adversary obtains or steals a user’s smart card and figures out the user’s password, the legitimate user can be easily impersonated. In section 1, an adversary is shown to compute the user’s password by using a smart card and public messages. Therefore, this scheme is critically deficient in that the adversary can be authenticated by the server without the user’s biometrics.

As described in Fig 6, the adversary can illegally extract all values including *K*_*i*_, *f*_*i*_, *e*_*i*_, and *EID*_*i*_ from the user’s smart card by monitoring the power consumption. It then computes *PW*_*i*_ using an off-line password attack computing *r*_*i*_ using *PW*_*i*_, *K*_*i*_, *f*_*i*_ as follows:

$$\begin{array}{c} r_{i}=h\left(PW_{i}⊕K\right)⊕f_{i} \end{array}$$

Even if *U*_*i*_ successfully executes the password change process, the adversary can still use these to impersonate a legal user, authenticate *S*_*i*_ without knowing the *B*_*i*_ values, and then compute normal authentication messages $EID_{i},M_{2}^{a},M_{3}^{a}$ using *r*_*i*_, *e*_*i*_, *EID*_*i*_ as follows:

$$\begin{array}{ccc} M_{1}=e_{i}⊕r_{i} & = & h\left(ID_{i}∥X_{s}\right),\;\text{generates}\;R_{c}^{a}\\ M_{2}^{a} & = & M_{1}⊕R_{c}^{a},\\ M_{3}^{a} & = & h(M_{1}∥R_{c}^{a}). \end{array}$$

After *S*_*i*_ receives the messages $EID_{i},M_{2}^{a}$, and $M_{3}^{a}$, then, *S*_*i*_ checks the legitimacy of the messages. However, *S*_*i*_ cannot distinguish between a normal *M*_9_ and an abnormal *M*_9_ because the adversary used accurate values like *h*(*ID*_*i*_‖*X*_*s*_), but the adversary normally computes *h*(*ID*_*i*_‖*X*_*s*_) using *r*_*i*_, *e*_*i*_.

Then, *S*_*i*_ sends the authentication messages 〈*EID*_*i*_, *M*_6_, *M*_7_〉 for *U*_*i*_. These are then used by the adversary to compute the next authentication message $M_{9}^{a}$ for *S*_*i*_ as follows,

$$\begin{array}{ccc} M_{8} & = & M_{6}⊕M_{1},\\ \text{if}\;M_{7} & = & h(M_{1}∥M_{8}),\\ M_{9}^{a} & = & h(M_{1}∥R_{c}^{a}∥M_{8}). \end{array}$$

Next, *S*_*i*_ checks that the received $M_{9}^{a}$ is the same as $M_{10}^{a}=h(M_{4}∥M_{5}^{a}∥R_{s})$. However, *S*_*i*_ cannot distinguish it from a normal *M*_9_ because the adversary uses accurate values like *M*_1_*h*(*ID*_*i*_‖*X*_*s*_) and $R_{c}^{a}$, which is used for $\langle EID_{i},M_{2}^{a},M_{3}^{a}\rangle$. Then, *S*_*i*_ accepts the login request for the adversary.

The adversary can be authenticated at *S*_*i*_ because he determined *EID*_*i*_, *e*_*i*_ and *r*_*i*_ through an off-line password attack, so *S*_*i*_ cannot distinguish between the adversary and a legitimate user. Since the user’s biometric information is not used during the login and authentication phase, *S*_*i*_ authenticates the adversary as a normal user. *S*_*i*_ cannot store and check the password and biometric information during the login and authentication phase due to the user’s privacy. Thus, to solve this problem, it is necessary to modify the way in which the authentication values *h*(*ID*_*i*_‖*X*_*s*_) are computed for the user. This value cannot be stored on the smart card, and it can only be computed by a legitimate user when the user simultaneously inputs the password and biometrics during the login and authentication phase.

### ID guessing attack

**Fig 8:**
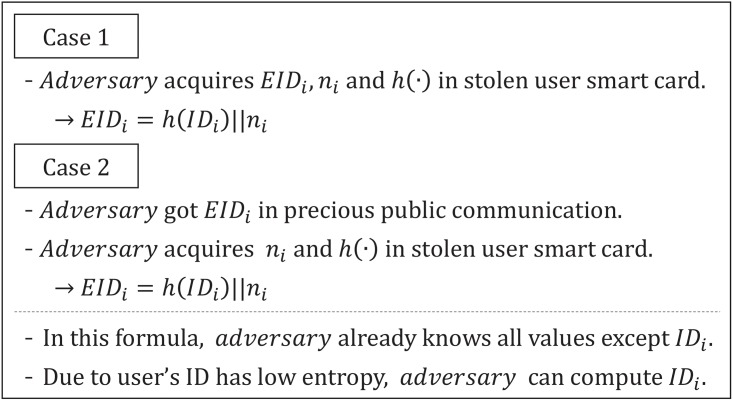
ID guessing attack on Cao and Ge’s authentication scheme.

Cao and Ge’s authentication scheme uses *EID* to protect the user’s *ID*_*i*_ in order to ensure user anonymity during public communication. However, the adversary can determine the user’s *ID*_*i*_ by using the user’s smart card and the public communication message *EID*_*i*_. Fig 8 describes in detail how to compute the user’s *ID*_*i*_ for Cao and Ge’s authentication scheme.

When an adversary obtains or steals a user’s smart card, he can extract *EID*_*i*_, *n*_*i*_ and *h*(⋅). Then, the adversary can compute the user *ID*_*i*_ from the formula *EID* = *h*(*ID*)‖*n*_*i*_ because he knows all values except for the *ID*_*i*_. In general, a user *ID*_*i*_ has a low entropy so the adversary is able to easily compute the user *ID*_*i*_. Basically, if an adversary fails to extract *EID*_*i*_ from the smart card, he can acquire *EID* from public communication. Therefore, even though the adversary extracts *n*_*i*_ and *h*(⋅) from the user’s smart card, he can determine the *ID*_*i*_ from *EID* = *h*(*ID*)‖*n*_*i*_. The user’s *ID*_*i*_ can be used for another attack, and therefore, the user’s *ID*_*i*_ needs to be protected using another value that the adversary cannot determine from the user’s smart card or from public communication.

### Vulnerability to a DoS attack

**Fig 9:**
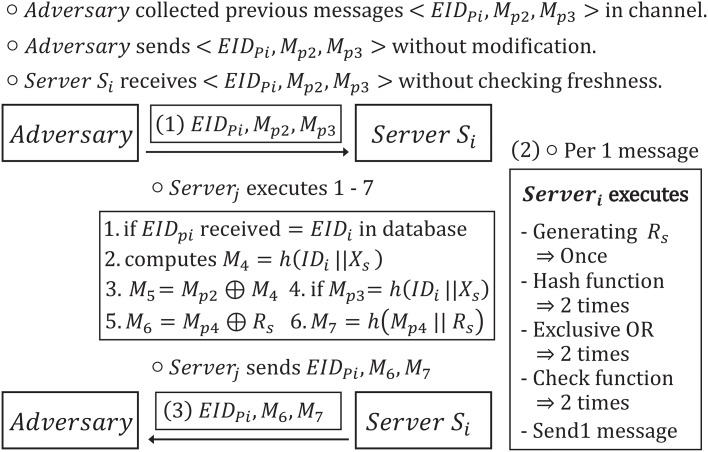
Vulnerability to a DoS attack on Cao and Ge’s authentication scheme.

A DoS attack is such where an adversary attempts to make a server or network resource become unavailable to prevent legitimate users from accessing the normal service. Although there are various ways to accomplish a DoS attack, the server’s system or configuration have to prepare for defenses against it. However, in Cao and Ge’s scheme, an adversary can execute a DoS attack without difficulty. Fig 9 describes the DoS attack for Cao and Ge’s authentication scheme.

An adversary can collect the previous messages 〈*EID*_*pi*_, *M*_*p*2_, *M*_*p*3_〉 from a legitimate user *U*_*i*_ and a server *S*_*i*_. Then, the adversary sends the messages to *S*_*i*_ without modification. The *S*_*i*_ unavoidably executes all operations of (2) and sends the (3) messages 〈*EID*_*pi*_, *M*_6_, *M*_7_〉 to the *U*_*i*_. This is the reason why *S*_*i*_ cannot verify the freshness of the (1) messages 〈*EID*_*pi*_, *M*_*p*2_, *M*_*p*3_〉. This operation involves the generation of a random nonce once, executing the hash function twice, calculating the exclusive-or operation twice, conducting the similarities checking function twice, and then, sending (3) messages 〈*EID*_*pi*_, *M*_6_, *M*_7_〉.

Therefore, the adversary can easily attempt to carry out a DoS attack targeting the server to see if he can obtain an intercepted number from a previous messages. Cao and Ge’s scheme does not check the freshness of an authentication message. Therefore, when an adversary sends previous authentication messages to *S*_*i*_, *S*_*i*_ cannot verify whether the received messages are current or not, and *S*_*i*_ is obligated to execute various operations. In order to defend against a DoS attack, this scheme needs to check the freshness of the messages by considering the timestamps.

### Lack of session key agreement

**Fig 10:**
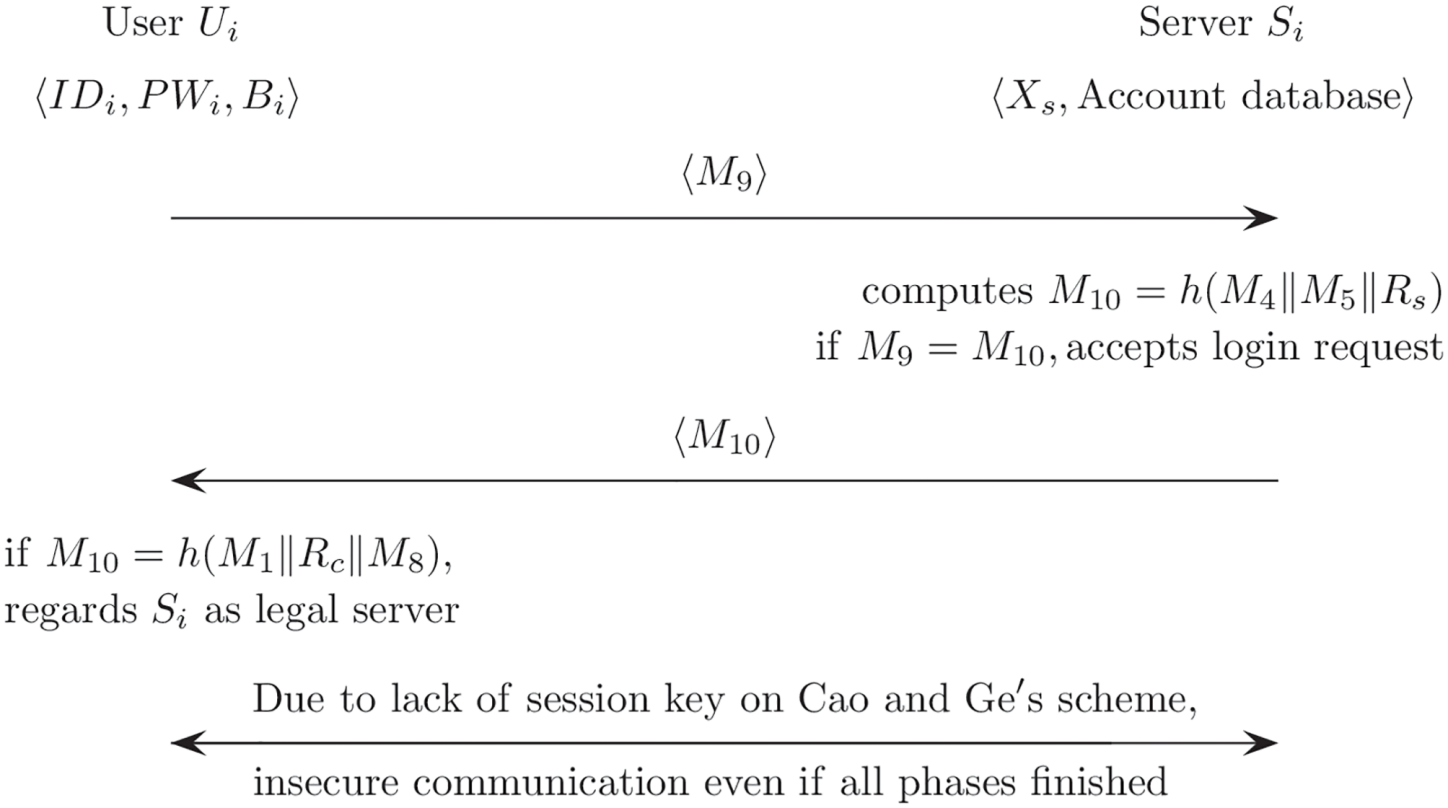
Lack of session key agreement on Cao and Ge’s authentication scheme.

In general, the session key refers to a symmetric key that is used to encrypt all messages in the communication session. Therefore, it can be computed and used for secure communications among communication members after successfully finishing the authentication phase. Fig 10 describes in detail the lack of session key agreement for Cao and Ge’s authentication scheme. As described in Fig 10, *U*_*i*_ and *S*_*i*_ finally authenticate each other using *M*_9_ and *M*_10_, and then they are accepted and regarded to be legal members. However, secure communication between *M*_9_ and *M*_10_ is not provided because these do not have a session key after all phases have finished. Therefore, it is necessary to modify the login and authentication phase to provide session key agreement. Moreover, to ensure the security of the scheme, the session key has to be changed for each session and must be secured against various forms of attack.

## Countermeasures

The reason why Cao and Ge’s scheme is vulnerable to the biometric recognition errors is that,

- even if the same user inputs his/her own biometrics to a scanner device, this device can generate slightly different outputs due to the general characteristics of the biometric information;
- the general hash function produces very large differences in the output data from slight differences in the input data.

Thus, a general hash function results in a legal user failing during the login phase when using his/her own biometrics. To prevent a biometric recognition error, we suggest modifying the registration phase from 〈*ID*_*i*_, *PW*_*i*_ ⊕ *K*, *B*_*i*_ ⊕ *K*〉 to

$$\begin{array}{c} \left\langle ID_{i},h\left(PW_{i}\right)⊕K,H\left(B_{i}\right)⊕K\right\rangle \end{array}$$

*H*(⋅) is a bio-hash function that produces consistent output for the same biometric information, even if the user’s biometric input is slightly different. So, during the login phase, the values need to be modified from *f*_*i*_ = *h*(*B*_*i*_ ⊕ *K*) to

$$\begin{array}{c} f_{i}=h\left(H\left(B_{i}\right)⊕K\right) \end{array}$$

However, by only modifying the scheme to use a bio-hash function, Cao and Ge’s authentication scheme is still vulnerable to the slow detection of a wrong password. This type of problem results from,

- the smart card not checking the user’s password during the login phase;
- the server can confirm whether a user inputs the wrong password and computes the wrong *M*_3_ during the authentication phase only after extensive computations;

Adding a password verification step during the login phase is suggested to solve the slow wrong password detection problem. Thus, the computations are modified for *f*_*i*_ from *f*_*i*_ from *f*_*i*_ = *h*(*H*(*B*_*i*_) ⊕ *K*) to

$$\begin{array}{c} f_{i}=h\left(ID_{i}⊕h\left(PW_{i}\right)⊕H\left(B_{i}\right)\right) \end{array}$$

However, even with the *f*_*i*_ modified above, an off-line password attack can still be carried out. This vulnerability is due to the fact that;

- an adversary can know and compute all formulas and values except for *PW*_*i*_;
- it is necessary to check *PW*_*i*_ with values, which the adversary cannot know and compute, such as *H*(*B*_*i*_);

Since we check the user’s password in *f*_*i*_, we suggest modifying *r*_*i*_ from *r*_*i*_ = *h*(*PW*_*i*_ ⊕ *K*) ⊕ *f*_*i*_ to

$$\begin{array}{c} r_{i}=h\left(H\left(B_{i}\right)⊕K\right)⊕f_{i} \end{array}$$

With such a modification, we can also defend against a user impersonation attack because the adversary cannot impersonate the user without the user’s password. In other words, the adversary cannot compute *r*_*i*_ without *PW*_*i*_ and then figure out *h*(*ID*_*i*_‖*X*_*s*_) to conduct a user impersonation attack due to the lack of a legal *M*_1_.

Next, the possible mechanism to eliminate the vulnerability in Cao and Ge’s scheme for an ID guessing attack is presented. This vulnerability is due to the fact that,

- the adversary can obtain the user’s *ID*_*i*_ from *EID*_*i*_ using the value *n*_*i*_ stored in the user’s smart card.
- Even if *EID*_*i*_ is a public communication message, Cao and Ge’s scheme does not provide sufficient protection for *EID*_*i*_.

To address to the problem on ID guessing attack, we suggest modifying *EID*_*i*_ from *EID*_*i*_ = *h*(*ID*_*i*_)‖*n*_*i*_ to

$$\begin{array}{c} EID_{i}=h(ID_{i}∥h\left(ID_{i}∥X_{s}\right)∥n_{i}) \end{array}$$

*h*(*ID*_*i*_‖*X*_*s*_) is not stored in a smart card, and it can be easily computed by *S*_*i*_. Even if the adversary knows *EID*_*i*_ and *n*_*i*_, he cannot compute *ID*_*i*_ from *EID*_*i*_ due to the ignorance on *h*(*ID*_*i*_‖*X*_*s*_).

However, with the modifications explained above, Cao and Ge’s scheme is still vulnerable to a DoS attack. The cause for this vulnerability on DoS attacks is that.

- *U*_*i*_ and *S*_*i*_ perform all operations without checking the freshness of the received authentication messages.
- Moreover, *S*_*i*_ unwillingly executes extensive computations per message before *S*_*i*_ discovers the fault of the received authentication message.

To address the vulnerability of the DoS attack, we suggest using timestamps (*T*_1_, *T*_2_, *T*_3_, *T*_4_) and adding them to the authentication messages. So we propose to modify the computations for *M*_3_, *M*_3_, *M*_3_, and *M*_10_ from *M*_3_ = *h*(*M*_1_‖*R*_*c*_), *M*_7_ = *h*(*M*_4_‖*R*_*s*_), *M*_9_ = *h*(*M*_1_‖*R*_*c*_‖*M*_8_), *M*_10_ = *h*(*M*_4_‖*M*_5_‖*R*_*s*_) to

$$\begin{array}{ccc} M_{3} & = & h(M_{1}∥R_{c}∥T_{1}),\\ M_{7} & = & h(M_{4}∥R_{s}∥T_{2}),\\ M_{9} & = & h(M_{1}∥R_{c}∥M_{8}∥T_{3}),\\ M_{10} & = & h(M_{4}∥M_{5}∥R_{s}∥T_{4}). \end{array}$$

In advance, all transmission messages need to include timestamps to check the freshness, such as from 〈*EID*_*i*_, *M*_2_, *M*_3_〉 to

$$\begin{array}{c} \left\langle EID_{i},M_{2},M_{3},T_{1}\right\rangle \end{array}$$

*T*_1_ and *M*_3_ are thus computed by a legal user, and the adversary cannot compute *M*_3_ without *T*_1_, which is current and matched with *M*_3_. So *S*_*i*_ can check the message freshness using *T*_1_, and *S*_*i*_ can verify the the message integrity and freshness by easily checking *M*_3_ = *h*(*M*_1_‖*R*_*c*_‖*T*_1_). In this manner, it is possibly to effectively prevent the DoS attack.

Finally, the problem regarding a lack of a session key is resolved by adding a session key agreement during the login and authentication phase. The session key needs to change for every session in order to enhance the security of the authentication scheme, so computing the session key agreement is proposed as follows;

$$\begin{array}{c} sk=h(h\left(ID_{i}∥X_{s}\right)∥R_{c}∥R_{s}|T_{3}∥T_{4}) \end{array}$$

For the session key agreement, *h*(*ID*_*i*_‖*X*_*s*_), *R*_*c*_ and *R*_*S*_ are computed only by the legal user and the server. *T*_3_ and *T*_4_ can be used to confirm the freshness of the session key. Therefore, this session key can change every session and can prevent various attacks.

## Security enhanced multi-factor biometric authentication scheme

To solve the problems inherent to Cao and Ge’s scheme, a security enhanced multi-factor biometric authentication scheme is proposed and divided into three phases: registration phase, password change phase, and login and authentication phase. Before our scheme is executed, *S*_*i*_ generates the server’s secure value *X*_*s*_ for security.

### Registration phase

**Fig 11:**
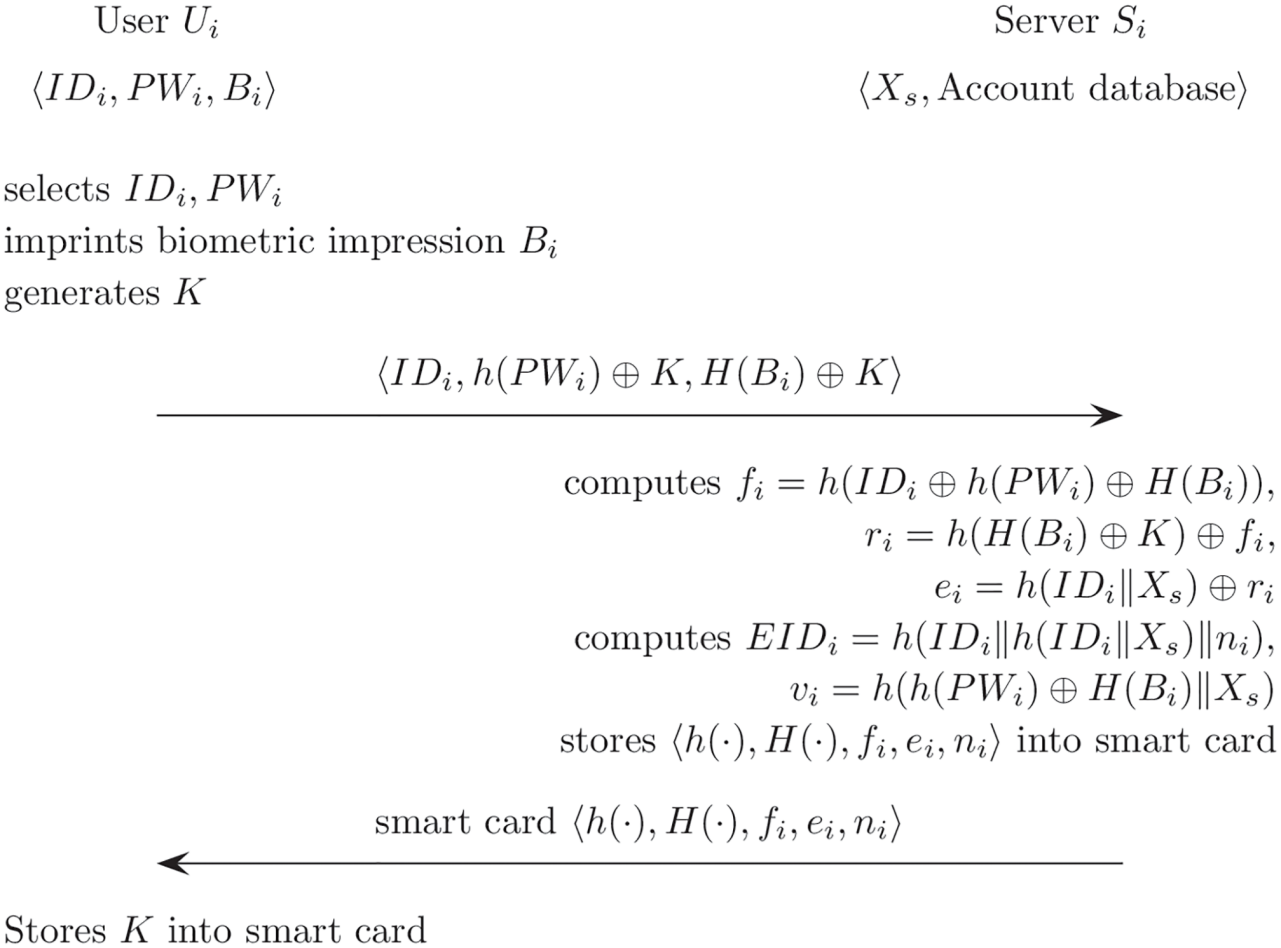
Registration phase for the proposed scheme.

The registration phase of the proposed scheme is described in Fig 11. *U*_*i*_ needs to perform the registration phase with *S*_*i*_ by using a secure channel.

(R1) *U*_*i*_ selects *ID*_*i*_, *PW*_*i*_; imprints the biometric impression *B*_*i*_; and generates *K*. *U*_*i*_ sends the identity *ID*_*i*_, *h*(*PW*_*i*_) ⊕ *K* using the general hash function, and *H*(*B*_*i*_) ⊕ *K* using bio-hash function to *S*_*i*_ through a secure channel.
(R2) After receiving these, *S*_*i*_ computes *f*_*i*_, *r*_*i*_, and *e*_*i*_ as follows;
  $$\begin{array}{ccc} f_{i} & = & h(ID_{i}⊕h\left(PW_{i}\right)⊕H\left(B_{i}\right)),\\ r_{i} & = & h\left(H\left(B_{i}\right)⊕K\right)⊕f_{i},\\ e_{i} & = & h\left(ID_{i}∥X_{s}\right)⊕r_{i}. \end{array}$$
(R3) Then, *S*_*i*_ creates an entry of database for the user *ID*_*i*_ and generates *n*_*i*_.
(R4) *S*_*i*_ computes *EID*_*i*_ and *v*_*i*_ as below, then *S*_*i*_ stores *EID*_*i*_, *ID*_*i*_, *n*_*i*_, *v*_*i*_ for *ID*_*i*_ as an entry in a database.
  $$\begin{array}{ccc} EID_{i} & = & h(ID_{i}∥h\left(ID_{i}∥X_{s}\right)∥n_{i}),\\ v_{i} & = & h(h\left(PW_{i}\right)⊕H\left(B_{i}\right)∥X_{s}). \end{array}$$
(R5) *S*_*i*_ sends a smart card to *U*_*i*_. The smart card contains 〈*h*(⋅), *H*(⋅), *f*_*i*_, *e*_*i*_, *n*_*i*_〉 through a secure channel. Then *U*_*i*_ stores *K* in the smart card.

### Password change phase

**Fig 12:**
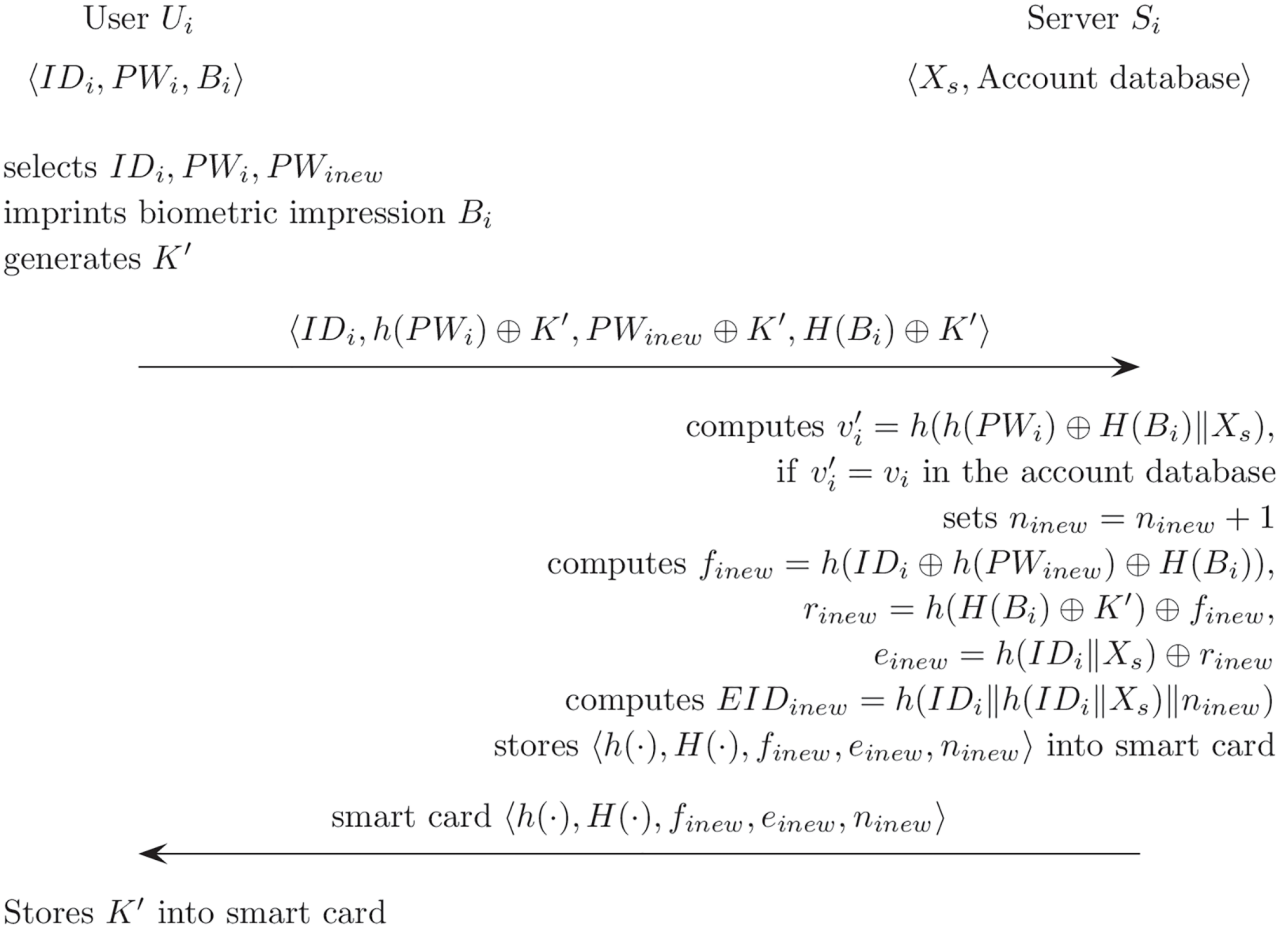
Password change phase for the proposed scheme.

For the proposed scheme, the password change phase is executed when *U*_*i*_ loses the smart card or wants to update the password. In order to change the password, *U*_*i*_ sends both the old password *PW*_*i*_ and new password *PW*_*inew*_. Fig 12 describes the password change phase for the proposed scheme.

(RR1) *U*_*i*_ selects and inputs *ID*_*i*_, *PW*_*i*_, and *PW*_*inew*_. *U*_*i*_ imprints its own biometric impression *B*_*i*_ and generates a new random value *K*′. Then, *U*_*i*_ submits 〈*ID*_*i*_, *h*(*PW*_*i*_) ⊕ *K*′, *h*(*PW*_*inew*_) ⊕ *K*′, *H*(*B*_*i*_) ⊕ *K*′〉 to *S*_*i*_ through a secure channel.
(RR2) After *S*_*i*_ receives these, *S*_*i*_ checks the database for the *ID*, and acquires the user’s data including *EID*_*i*_, *ID*_*i*_, *n*_*i*_, and *v*_*i*_. Then, *S*_*i*_ computes $v_{i}^{\prime }=h\left(h\left(PW_{i}\right)⊕H\left(B_{i}\right)∥X_{s}\right)$ and compares $v_{i}^{\prime }$ with *v*_*i*_ in the database.
(RR3) *S*_*i*_ sets *n*_*inew*_ = *n*_*i*_ + 1. Then, *S*_*i*_ carries out the computations as follows:
  $$\begin{array}{ccc} f_{inew} & = & h(ID_{i}⊕h\left(PW_{inew}\right)⊕H\left(B_{i}\right)),\\ r_{inew} & = & h\left(H\left(B_{i}\right)⊕K^{\prime }\right)⊕f_{inew},\\ e_{inew} & = & h\left(ID_{i}∥X_{s}\right)⊕r_{inew}. \end{array}$$
(RR4) *S*_*i*_ computes *EID*_*inew*_ = *h*(*ID*_*i*_‖*h*(*ID*_*i*_‖*X*_*s*_)‖*n*_*inew*_), then *S*_*i*_ stores *EID*_*inew*_, *ID*_*i*_, *n*_*inew*_ for *ID*_*i*_ to the entry of database.
(RR5) *S*_*i*_ sends a new smart card to *U*_*i*_ that contains 〈*h*(⋅), *H*(⋅), *f*_*inew*_, *e*_*inew*_, *n*_*inew*_〉 by using a secure channel. Then *U*_*i*_ stores a new *K*′ in the smart card.

### Login and authentication phase

**Fig 13:**
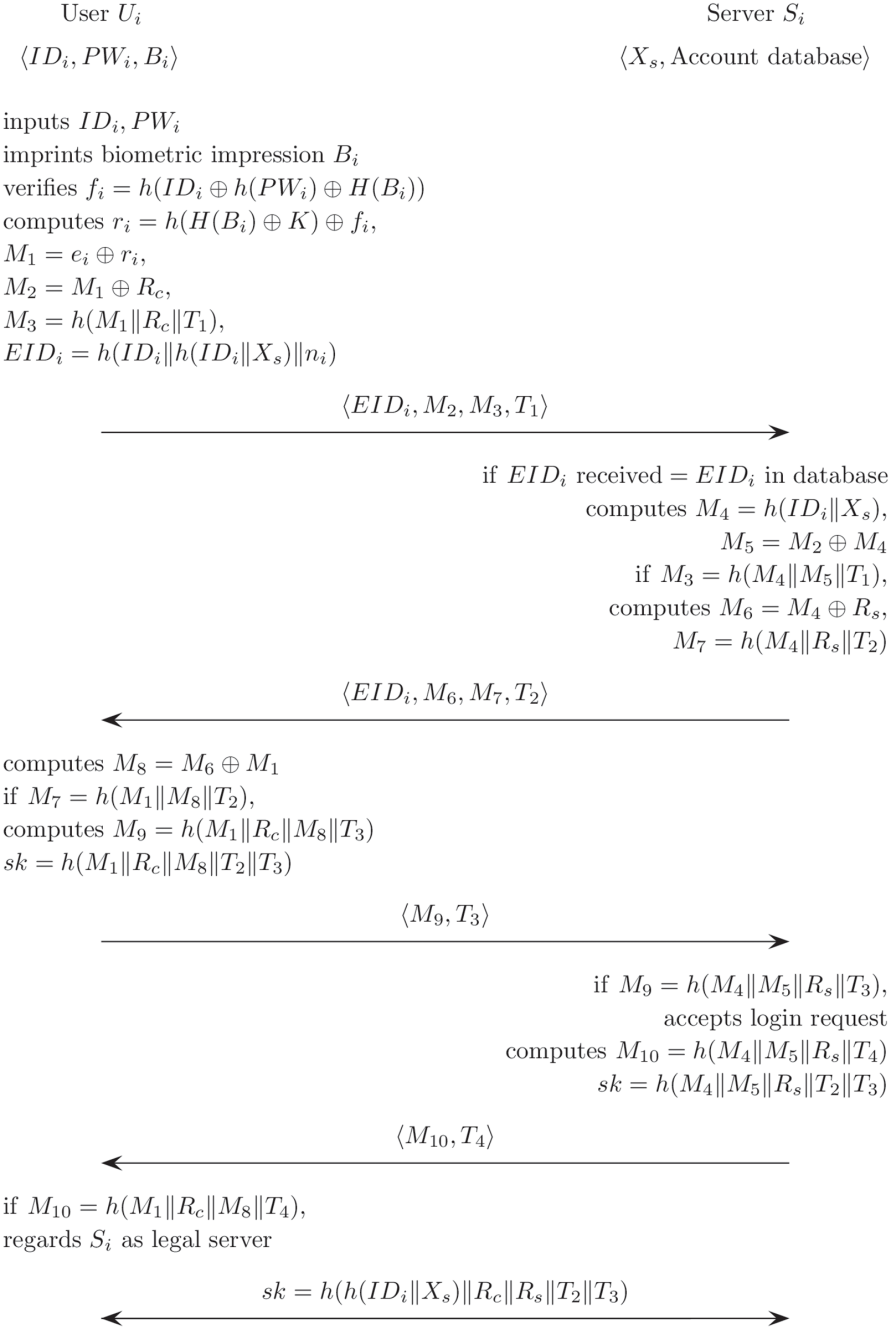
Login and authentication phase for the proposed scheme.

Fig 13 describes the login and authentication phase for the proposed scheme. *U*_*i*_ executes the following steps when *U*_*i*_ wants to authenticate a remote *S*_*i*_. In this phase, the smart card checks the legitimacy of the user using *ID*_*i*_, *PW*_*i*_ and *B*_*i*_.

(L1) *U*_*i*_ inputs the *ID*_*i*_ and *PW*_*i*_; *U*_*i*_ imprints *B*_*i*_ using a biological feature extraction device; computes *h*(*PW*_*i*_) using the general hash function and *H*(*B*_*i*_) using the bio-hash function. Then, the smart card computes *f*_*i*_, and is verified as follows,
  $$\begin{array}{ccc} f_{i} & = & h(ID_{i}⊕h\left(PW_{i}\right)⊕H\left(B_{i}\right)). \end{array}$$
(L2) If they are the same, *U*_*i*_ generates the current timestamp *T*_*i*_ and a random number *R*_*c*_. Then, *U*_*i*_ computes *r*_*i*_, *M*_1_, *M*_2_, *M*_3_, *EID*_*i*_ using the user’s input values and the smart card storing values as follows;
  $$\begin{array}{ccc} r_{i} & = & h\left(H\left(B_{i}\right)⊕K\right)⊕f_{i},\\ M_{1} & = & e_{i}⊕r_{i},\\ M_{2} & = & M_{1}⊕R_{c},\\ M_{3} & = & h(M_{1}∥R_{c}∥T_{1}),\\ EID_{i} & = & h(ID_{i}∥h\left(ID_{i}∥X_{s}\right)∥n_{i}). \end{array}$$
(L3) *U*_*i*_ sends the login request message 〈*EID*_*i*_, *M*_2_, *M*_3_, *T*_1_〉 to *S*_*i*_.

The server *S*_*i*_ executes the authentication phase when the message is received.

(A1) *S*_*i*_ checks that the *EID*_*i*_ satisfies the original format.
(A2) If the *ID*_*i*_ is valid when compared with the user’s entry in the database in *S*_*i*_, *S*_*i*_ computes *M*_4_ and *M*_5_, and then verifies *M*_3_ as follows,
  $$\begin{array}{ccc} M_{4} & = & h(ID_{i}∥X_{s}),\\ M_{5} & = & M_{2}⊕M_{4},\\ M_{3} & = & h(M_{4}∥M_{5}∥T_{1}). \end{array}$$
(A3) If *M*_3_ is accurate, *S*_*i*_ generates the current timestamp *T*_2_ and computes *M*_6_ and *M*_7_. Then, *S*_*i*_ sends the message 〈*EID*_*i*_, *M*_6_, *M*_7_, *T*_2_〉 to *U*_*i*_.
  $$\begin{array}{ccc} M_{6} & = & M_{4}⊕R_{s},\\ M_{7} & = & h(M_{4}∥R_{s}∥T_{2}). \end{array}$$
(A4) *U*_*i*_ computes *M*_8_ = *M*_6_ ⊕ *M*_1_ and verifies whether *M*_7_ = *h*(*M*_1_‖*M*_8_‖*T*_2_) or not. If they are equal, *U*_*i*_ generate a timestamp *T*_3_ and computes *M*_9_. Then *U*_*i*_ computes *sk* as follows.
  $$\begin{array}{ccc} M_{9} & = & h(M_{1}∥R_{c}∥M_{8}∥T_{3}),\\ sk & = & h(M_{1}∥R_{c}∥M_{8}∥T_{2}∥T_{3}). \end{array}$$
(A5) *U*_*i*_ sends the message 〈*M*_9_, *T*_3_〉 to *S*_*i*_.
(A6) After receiving 〈*M*_9_〉, *S*_*i*_ verifies that *M*_9_ is equal to *h*(*M*_4_‖*M*_5_‖*R*_*s*_‖*T*_3_) and then accepts the user’s login request. *S*_*i*_ computes *M*_10_ = *h*(*M*_4_‖*M*_5_‖*R*_*s*_‖*T*_4_) and *sk*. Then, *S*_*i*_ sends 〈*M*_10_, *T*_4_〉 to *U*_*i*_.
  $$\begin{array}{c} sk=h(M_{4}∥M_{5}∥R_{s}∥T_{2}∥T_{3}) \end{array}$$
(A7) After receiving 〈*M*_10_, *T*_4_〉, *U*_*i*_ verifies that *M*_10_ is equal to *h*(*M*_1_‖*R*_*c*_‖*M*_8_‖*T*_4_) and regards *S*_*i*_ as a legal server.
(A8) Therefore, *U*_*i*_ and *S*_*i*_ share the same session key after all phases have finished.
  $$\begin{array}{c} sk=h(h\left(ID_{i}∥X_{s}\right)∥R_{c}∥R_{s}∥T_{2}∥T_{3}) \end{array}$$

## Analysis

Several analyses were carried out to confirm that the proposed scheme with a bio-hash function improves the security of the authentication process. Ding Wang *et al*. analyzed various smart-card-based password authentication methods and introduced a good solution using the principle of the security-usability trade-off to prevent off-line password attacks. Ding Wang *et al*. proposed that a fuzzy verifier can resolve the trade-off between the security requirement of resistance to smart card loss attack and the usability goal of a local password change [35–37].

In this paper, the proposed scheme uses a bio-hash function, which is similar to a fuzzy verifier to secure the system against various types of off-line guessing attacks. The proposed scheme is investigated by conducting a security analysis, a formal analysis, and an efficiency analysis. Then, the proposed scheme is compared to other authentication schemes, including Cao and Ge’s scheme. We follow a security definition with strong secret values (*B*_*i*_, *x*) with a high entropy that cannot be guessed in polynomial time and a secure one-way hash function *y* = *h*(*x*). Given *x* to compute *y* is easy but *y* to compute *x* is much more difficult.

### Security analysis

This section describes a security analysis to confirm the security of the proposed scheme.

1. [**Replay attack**] In the proposed scheme, even if an adversary intercepts the messages like 〈*EID*_*i*_, *M*_2_, *M*_3_, *T*_1_〉 and 〈*M*_9_, *T*_3_〉 over public communication and replays 〈*EID*_*i*_, *M*_2_, *M*_3_, *T*_1_〉 to *S*_*i*_, he cannot authenticate with *S*_*i*_. First, it is hard for the adversary to respond within the allowable time for timestamp *T*_1_, and even though the adversary passes the time limit, he cannot execute the appropriate response for 〈*EID*_*i*_, *M*_6_, *M*_7_, *T*_2_〉. The adversary has only the previous 〈*M*_9_, *T*_3_〉, which is not appropriate for the response because he cannot know the new *R*_*c*_. Only a legal user can know the new *R*_*c*_ using *h*(*ID*_*i*_‖*X*_*s*_). Therefore, the adversary cannot succeed in the replay attack due to the timestamps and the lack of knowledge of *h*(*ID*_*i*_‖*X*_*s*_) [53].
2. [**Server masquerading attack**] If an adversary wants to masquerade as a legal server, he has to send the appropriate response to the user’s request. When the user sends 〈*M*_9_, *T*_3_〉 to the adversary, he has to compute the appropriate 〈*M*_10_, *T*_4_〉 to look like a legal server. However, if the adversary wants to compute 〈*M*_10_, *T*_4_〉 using *M*_9_, *T*_3_ and *T*_4_, he has to know the *R*_*c*_ and *h*(*ID*‖*X*_*s*_). Only a legal server can compute 〈*M*_10_, *T*_4_〉 because the legal server stored *X*_*s*_ and *R*_*c*_ in the database and the adversary cannot know them. Therefore, the adversary cannot succeed in masquerading as a legal server.
3. [**Mutual authentication**] Mutual authentication means that a user and a server authenticate each other. In the proposed scheme, *U*_*i*_ and *S*_*i*_ authenticate each other by checking for a mutual random number, which is possible for a legal user and server because only they know *h*(*ID*_*i*_‖*X*_*s*_). Specifically, *S*_*i*_ authenticates *U*_*i*_ according to the 〈*M*_9_, *T*_3_〉 that is received because only a legal *U*_*i*_ can compute *M*_9_ using *S*_*i*_’s *M*_6_. *U*_*i*_ authenticates *S*_*i*_ by 〈*M*_10_, *T*_4_〉, and only the server can compute *M*_10_ from 〈*M*_9_, *T*_3_〉 because only the legal server can know the user’s random number *R*_*c*_ using *h*(*ID*_*i*_‖*X*_*s*_), *R*_*c*_ = *M*_2_ ⊕ *h*(*ID*_*i*_‖*X*_*s*_) [54].
4. [**Biometric recognition error**] The proposed scheme uses a bio-hash function to prevent a biometric recognition error. Cao and Ge’s scheme uses a general hash function to verify the user’s biometrics, so a biometric recognition error happens as a result of the general hash function’s behavior. However, the proposed scheme uses a bio-hash function for the user’s biometric information because the bio-hash function provides consistent output for the same biometric information, even when a user’s biometrics are input a little differently.
5. [**Slow wrong password detection**] Unlike Cao and Ge’s scheme, the proposed scheme can check the user’s password during the login phase. Therefore, it is possible to verify whether or not the user has input an accurate password. In the proposed scheme, when a user wants to login and authenticate on a server, he inputs his own *ID*_*i*_, *PW*_*i*_, and *B*_*i*_. Using these, the smart card computes *f*_*i*_ = *h*(*ID*_*i*_ ⊕ *h*(*PW*_*i*_) ⊕ *H*(*B*_*i*_)) and computes it with *f*_*i*_, which is stored in a smart card. If the user inputs the wrong password, the computed *f*_*i*_ and stored *f*_*i*_ will be different, so the user can immediately know whether he needs to input the correct password again.
6. [**Off-line password attack**] An adversary can extract all information stored in the user’s smart card by using a side-channel attack, such as by physically monitoring the power consumption. However, in the proposed scheme, the user’s password is always used with the user’s *ID*_*i*_ and the biometrics information *H*(*B*_*i*_) like *f*_*i*_ = *h*(*ID*_*i*_ ⊕ *h*(*PW*_*i*_) ⊕ *H*(*B*_*i*_). The user’s *ID*_*i*_ is protected by *EID*_*i*_ = *h*(*ID*_*i*_‖*h*(*ID*‖*X*_*s*_)‖*n*_*i*_). Moreover, *B*_*i*_ has a high entropy, so the adversary cannot carry out the computation. Therefore, even if the adversary extracts *f*_*i*_ using a side channel attack, he cannot compute the user’s password because he cannot know both *ID*_*i*_ and *H*(*B*_*i*_).
7. [**User impersonation attack**] To successfully carry out a user impersonation attack, an adversary needs to know the user’s *h*(*ID*_*i*_‖*X*_*i*_). In order to compute *h*(*ID*_*i*_‖*X*_*i*_), the adversary must know *r*_*i*_ using *f*_*i*_ and *e*_*i*_; *f*_*i*_ = *h*(*ID*_*i*_ ⊕ *h*(*PW*_*i*_) ⊕ *H*(*B*_*i*_)), *e*_*i*_ = *h*(*ID*_*i*_‖*X*_*s*_) ⊕ *r*_*i*_.
  $$\begin{array}{c} r_{i}=h\left(H\left(B_{i}\right)⊕K\right)⊕f_{i}. \end{array}$$
  However, *r*_*i*_ is protected by *h*(*H*(*B*_*i*_) ⊕ *K*), and the adversary cannot know *H*(*B*_*i*_). Therefore the proposed scheme prevents a user impersonation attack.
8. [**ID guessing attack**] Unlike for *EID*_*i*_ = *h*(*ID*_*i*_‖*n*_*i*_) in Cao and Ge’s scheme, the proposed scheme uses *EID*_*i*_ = *h*(*ID*_*i*_‖*h*(*ID*_*i*_‖*X*_*s*_)‖*n*_*i*_) to protect the user’s *ID*_*i*_. An adversary can extract *n*_*i*_ from the smart card and can obtain *EID*_*i*_ from public communications. However, if *h*(*ID*_*i*_‖*X*_*s*_) is not stored in a smart card and can only be easily computed by a legal *U*_*i*_ and *S*_*i*_, then the adversary cannot compute *h*(*ID*_*i*_‖*X*_*s*_). Therefore, even if the adversary knows *EID*_*i*_ and *n*_*i*_, he cannot compute *ID*_*i*_ from *EID*_*i*_ due to the ignorance of *h*(*ID*_*i*_‖*X*_*s*_).
9. [**Vulnerability to a DoS attack**] The proposed scheme checks the freshness of all messages using a timestamp *T*_1_, *T*_2_, *T*_3_, *T*_4_, so it is useless for an adversary to send the previous messages to the server. Moreover, *U*_*i*_ and *S*_*i*_ authenticate each other using the messages including current timestamps; *M*_3_ = *h*(*M*_1_‖*R*_*c*_‖*T*_1_), *M*_7_ = *h*(*M*_4_‖*R*_*s*_‖*T*_2_), *M*_9_ = *h*(*M*_1_‖*R*_*c*_‖*M*_8_‖*T*_3_), *M*_10_ = *h*(*M*_4_‖*M*_5_‖*R*_*s*_‖*T*_4_). For example, *S*_*i*_ can check the freshness and legality of *M*_3_ because *M*_3_ and the timestamp *T*_1_ do not match, even if the adversary sends the previous *M*_3_ with the current timestamp. Therefore, the proposed scheme is more secure than Cao and Ge’s authentication scheme against a DoS attack.
10. [**Lack of session key agreement**] Cao and Ge’s authentication scheme does not provide a session key agreement, so it cannot establish secure communications with an encryption after all phases have finished. To resolve the problem of the lack of a session key, a session key agreement is provided during the login and authentication phase. In order to share the session key *sk* = *h*(*h*(*ID*_*i*_‖*X*_*s*_)‖*R*_*c*_‖*R*_*s*_|*T*_2_‖*T*_3_). *h*(*ID*_*i*_‖*X*_*s*_), *R*_*c*_ and *R*_*S*_ are computed only by a legal *U*_*i*_ and *S*_*i*_. *T*_2_ and *T*_3_ can be used to confirm the freshness of the session key, and the session key of the proposed scheme can be changed at every session to prevent various forms of attack [55].

Table 2 shows a comparison of the security analysis for various multi-factor authentication schemes, including our proposed scheme [14, 38, 39, 50, 56–58].

**Table 2:** Security analysis for various authentication schemes.

Attack resistance	[14]	[38]	[39]	[50]	[56]	[57]	[58]	Ours
Replay attack	O	O	O	O	O	O	O	O
Server masquerading attack	X	X	X	O	X	O	X	O
Mutual authentication	O	O	O	O	X	X	X	O
Biometric recognition error	X	X	X	X	X	X	X	O
Slow wrong password detection	X	X	X	X	O	O	O	O
Off-line password attack	X	O	O	X	X	X	X	O
User impersonation attack	X	O	O	X	X	X	X	O
ID guessing attack	X	X	X	X	X	X	X	O
Vulnerability to a DoS attack	X	X	X	X	X	O	X	O
Lack of session key agreement	X	X	X	X	O	O	O	O

### Formal analysis

BAN logic (Burrows-Abadi-Needham logic) was introduced by Burrows M, and it has consistently drawn attention due to the simplicity and straightforwardness of the analysis of authentication schemes, and in this section, we analyze the proposed scheme using BAN-logic with symbols *P* and *Q* representing principals and *X* and *Y* representing statements. The main notation of the logic is presented in BAN’s paper and main inference rules. The analysis of an authentication scheme using the BAN-logic tool consists of four steps, and the formal analysis of the security of the proposed scheme is described as follows. The analysis shows that a session key can be generated correctly between the communicating parties during authentication. First, the notation of BAN logic being used in this scheme is introduced [59–62].

- *P*∣≡ *X*: The principal *P* believes statement *X*. This means that *P* believes that in the current run of the scheme, the statement *X* is true.
- *P* ⊲ *X*: The principal *P* sees the statement *X*, which means that *P* has received a message containing *X*.
- *P*∣∼*X*: The principal *P* once said the statement *X*, which means that *P*∣≡*X* when *P* sent it.
- *P* ⇒ *X*: The principal *P* has jurisdiction over statement *X*. This means that *P* has complete control on the formula *X*.
- ♯(X): The formula *X* is fresh. This means that formula *X* has not been used before.
- $P|\equiv Q\overset{K}{↔}P$: *P* believes that the principal *P* and *Q* communicate with each other using *K*.
- $P\overset{K}{↔}X$: *K* is shared secret information between *P* and *Q*. The secret key *K* is known only to *P* and *Q*, and *K* is a secret between both parties.
- {*X*}_*K*_: The formula *X* is encrypted using the secret key *K*.
- 〈*X*〉_*K*_: The formula *X* is combined including the secret key *K*.
- (*X*)_*K*_: The formula *X* is hashed including the secret key *K*.
- *sk*: The session key used in the current session.

To describe the logical postulates of BAN logic, we present the following rules:

1. Message-meaning rule: $\frac{P|\equiv P\overset{K}{↔}Q,P⊲{\left(X\right)}_{K}}{P|\equiv Q|∼X}$: if the principal *P* believes he/she shares the secret key *K* with *Q*, *P* sees the statement *X* hashed to include the *K*. Then *P* believes that *Q* once said *X*.
2. Nonce-verification rule: $\frac{P|\equiv \#(X),P|\equiv Q|∼X}{P|\equiv Q|\equiv X}$: if principal *P* believes that *X* is fresh and *P* believes *Q* once said *X*, then *P* believes that *Q* believes *X*.
3. The belief rule: $\frac{P|\equiv X,P|\equiv Y}{P|\equiv (X,Y)}$: if principal *P* believes both *X* and *Y*, then *P* believes (*X*, *Y*).
4. Freshness-conjuncatenation rule: $\frac{P|\equiv \#(X)}{P|\equiv \#(X,Y)}$: if principal *P* believes *X* is fresh, then *P* believes (*X*, *Y*) is fresh.
5. Jurisdiction rule: $\frac{P|\equiv Q|\Rightarrow X,P|\equiv Q|\equiv X}{P|\equiv X}$: if principal *P* believes that *Q* has jurisdiction over *X* and *P* believes that *Q* believes *X*, then *P* believes *X*.

According to the analytic procedures of BAN logic and using previously described logical postulates, the proposed scheme needs to satisfy the following goals:

- Goal 1: $S|\equiv (U\overset{sk}{↔}S)$.
- Goal 2: $U|\equiv (U\overset{sk}{↔}S)$.
- Goal 3: $S|\equiv U|\equiv (U\overset{sk}{↔}S)$.
- Goal 4: $U|\equiv S|\equiv (U\overset{sk}{↔}S)$.

The generic type of proposed scheme is as follows:

- Message 1.
  *U* → *S*: *h*(*ID*_*i*_‖*h*(*ID*_*i*_‖*X*_*s*_)‖*n*_*i*_), *h*(*ID*_*i*_‖*X*_*s*_) ⊕ *R*_*c*_, *h*(*h*(*ID*_*i*_‖*X*_*s*_)‖*R*_*c*_‖*T*_1_), *T*_1_
- Message 2.
  *S* → *U*: *h*(*ID*_*i*_‖*h*(*ID*_*i*_‖*X*_*s*_)‖*n*_*i*_), *h*(*ID*_*i*_‖*X*_*s*_) ⊕ *R*_*s*_, *h*(*h*(*ID*_*i*_‖*X*_*s*_)‖*R*_*s*_‖*T*_2_), *T*_2_
- Message 3.
  *U* → *S*: *h*(*h*(*ID*_*i*_‖*X*_*s*_)‖*R*_*c*_‖*R*_*s*_‖*T*_3_), *T*_3_
- Message 4.
  *S* → *U*: *h*(*h*(*ID*_*i*_‖*X*_*s*_)‖*R*_*c*_‖*R*_*s*_‖*T*_4_), *T*_4_

The idealized form of proposed scheme is as follows:

- Message 1. *U* → *S*: (*ID*_*i*_, *n*_*i*_)_*h*(*ID*_*i*_‖*X*_*s*_)_, 〈*R*_*c*_〉_*h*(*ID*_*i*_‖*X*_*s*_)_, (*R*_*c*_, *T*_1_)_*h*(*ID*_*i*_‖*X*_*s*_)_, *T*_1_
- Message 2. *S* → *U*: (*ID*_*i*_, *n*_*i*_)_*h*(*ID*_*i*_‖*X*_*s*_)_, 〈*R*_*s*_〉_*h*(*ID*_*i*_‖*X*_*s*_)_, (*R*_*s*_, *T*_2_)_*h*(*ID*_*i*_‖*X*_*s*_)_, *T*_2_
- Message 3. *U* → *S*: ${\left(R_{c},R_{s},T_{3}\right)}_{h(ID_{i}∥X_{s})},T_{3},U\overset{sk}{↔}S$
- Message 4. *S* → *U*: ${\left(R_{c},R_{s},T_{4}\right)}_{h(ID_{i}∥X_{s})},T_{4},U\overset{sk}{↔}S$

We make the following assumptions for the initial state of the protocol to analyze the proposed protocol:

- A1: *U* ∣≡ ♯(*T*_1_)
- A2: *S* ∣≡ ♯(*T*_2_)
- A3: *U* ∣≡ ♯(*T*_3_)
- A4: *S* ∣≡ ♯(*T*_4_)
- A5: $U|\equiv (U\overset{h(ID_{i}∥X_{s})}{↔}S)$
- A6: $S|\equiv (U\overset{h(ID_{i}∥X_{s})}{↔}S)$
- A7: $U|\equiv S\Rightarrow (U\overset{sk}{↔}S)$
- A8: $S|\equiv U\Rightarrow (U\overset{sk}{↔}S)$

The idealized form of the proposed protocol based on BAN logic rules and assumptions is analyzed. The main proofs are described as follows.

According to Message 3, we could obtain:

- S1: $S⊲\{{\left(R_{c},R_{s},T_{3}\right)}_{h(ID_{i}∥X_{s})},T_{3},U\overset{sk}{↔}S\}$
  According to the assumption A6 and the message meaning rule, we obtain:
- S2: $S|\equiv U|∼\{{\left(R_{c},R_{s},T_{3}\right)}_{h(ID_{i}∥X_{s})},T_{3},U\overset{sk}{↔}S\}$
  According to the assumption A3 and the freshness conjuncatenation rule, we can obtain:
- S3: $S|\equiv ♯\{{\left(R_{c},R_{s},T_{3}\right)}_{h(ID_{i}∥X_{s})},T_{3},U\overset{sk}{↔}S\}$
  According to the assumption S2, S3 and the nonce verification rule, we obtain:
- S4: $S|\equiv U|\equiv \{{\left(R_{c},R_{s},T_{3}\right)}_{h(ID_{i}∥X_{s})},T_{3},U\overset{sk}{↔}S\}$
  According to S4, we apply the belief rule, we obtain:
- S5: $S|\equiv U|\equiv (U\overset{sk}{↔}S)$, We satisfy (**Goal 3**. $S|\equiv U|\equiv (U\overset{sk}{↔}S\;)$
  According to the assumption A8, S5 and the jurisdiction rule, we can obtain the conclusion as follows:
- S6: $S|\equiv (U\overset{sk}{↔}S)$, We satisfy (**Goal 1**. $S|\equiv (U\overset{sk}{↔}S)\;)$

According to the message 4, we obtain:

- S7: $U⊲\{{\left(R_{c},R_{s},T_{4}\right)}_{h(ID_{i}∥X_{s})},T_{4},U\overset{sk}{↔}S\}$
  According to the assumption A5 and the message meaning rule, we obtain:
- S8: $U|\equiv S|∼\{{\left(R_{c},R_{s},T_{4}\right)}_{h(ID_{i}∥X_{s})},T_{4},U\overset{sk}{↔}S\}$
  According to the assumption A4 and the freshness conjuncatenation rule, we obtain:
- S9: $U|\equiv ♯\{{\left(R_{c},R_{s},T_{4}\right)}_{h(ID_{i}∥X_{s})},T_{4},U\overset{sk}{↔}S\}$
  According to assumption S8, S9 and the nonce verification rule, we obtain:
- S10: $U|\equiv S|\equiv \{{\left(R_{c},R_{s},T_{4}\right)}_{h(ID_{i}∥X_{s})},T_{4},U\overset{sk}{↔}S\}$
  According to S10, we apply the belief rule, we obtain:
- S11: $U|\equiv S|\equiv (U\overset{sk}{↔}S)$, We satisfy (**Goal 4**. $U|\equiv S|\equiv (U\overset{sk}{↔}S\;)$
  According to the assumption A7, S11 and the jurisdiction rule, we can obtain the conclusion as follows:
- S12: $U|\equiv (U\overset{sk}{↔}S)$, We satisfy (**Goal 2**. $U|\equiv (U\overset{sk}{↔}S)\;)$

### Efficiency analysis

The computational costs of the modified scheme and others are calculated in Table 3. *T*_*h*_ stands for the computation time of the hash function while the computation time for the exclusive OR operation *T*_*XOR*_ does not appear in the table because it can be ignored when compared to *T*_*h*_.

**Table 3:** Computational costs.

Phases	[14]	[38]	[39]	[50]	[56]	[57]	[58]	Ours
Registration phase	3 *T*_*h*_	3 *T*_*h*_	3 *T*_*h*_	7 *T*_*h*_	5 *T*_*h*_	7 *T*_*h*_	4 *T*_*h*_	7 *T*_*h*_
Login phase	2 *T*_*h*_	3 *T*_*h*_	2 *T*_*h*_	4 *T*_*h*_	11 *T*_*h*_	4 *T*_*h*_	4 *T*_*h*_	4 *T*_*h*_
Authentication phase	5 *T*_*h*_	6 *T*_*h*_	8 *T*_*h*_	7 *T*_*h*_	4 *T*_*h*_	11 *T*_*h*_	13 *T*_*h*_	9 *T*_*h*_

According to the results obtained in [63], *T*_*h*_ needs a time of about 0.20 ms (*T*_*h*_ ≈ 0.20 ms) on a system using 3.0 GB RAM with a Pentium IV 3.2 GHz processor. Table 4 shows the efficiency for various authentication scheme obtained through a simulation.

**Table 4:** Efficiency simulation.

Authentication scheme	[14]	[38]	[39]	[50]	[56]	[57]	[58]	Ours
Execution time (millisecond)	2.0	2.4	2.6	3.6	4.0	4.4	4.2	4.0

As shown in Tables 3 and 4, the modified scheme requires a slightly higher computational cost than the others, but mainly in the registration phase [38–40, 50]. However, the modified scheme can provide all security properties shown in Table 2.

## Conclusions

This paper discusses possible attacks for Cao and Ge’s authentication scheme, and a modified scheme is proposed to improve security and protect against various attacks. A security analysis and efficiency analysis are carried out to compare the results of the modified scheme to those of other schemes. In addition, the modified scheme is verified by conducting a formal security analysis using BAN-logic. The results indicate that the modified scheme has a slightly higher computational cost but that it is more secure than some of the other related schemes. The proposed scheme uses a bio-hash function for multi-factor biometric authentication to improve security. We also intend to conduct further studies on verification techniques, such as a fuzzy verifier and bio-hash function, to resolve the security-usability trade-off.

## References

[1] Choi Y, Nam J, Lee D, Kim J, Jung J, and Won D. Security Enhanced Anonymous Multiserver Authenticated Key Agreement Scheme Using Smart Cards and Biometrics. The Scientific World Journal. 2014 10.1155/2014/281305 PMC4170879 25276847

[2] Huang H, and Cao Z. IDOAKE: strongly secure ID-based one-pass authenticated key exchange protocol. Security and Communication Networks. 2013: 1153–1161.

[3] Rivest RL, Shamir A and Adleman L. A method for obtaining digital signatures and public-key cryptosystems. Communications of the ACM. 1978: 120–126. 10.1145/359340.359342

[4] Lamport L. Password authentication with insecure communication. Communications of the ACM. 1981;24(11); 770–772. 10.1145/358790.358797

[5] ElGamal T. (1985, 1). A public key cryptosystem and a signature scheme based on discrete logarithms In Advances in cryptology. Springer Berlin Heidelberg 10–18.

[6] Choi Y, Lee D, Kim J, Jung J, Nam J, and Won D. Security enhanced user authentication protocol for wireless sensor networks using elliptic curves cryptography. Sensors. 2014;14(6): 10081–10106. 10.3390/s140610081 24919012 PMC4118368

[7] Koblitz N. Elliptic curve cryptosystems. Mathematics of computation. 1987;48(177): 203–209. 10.1090/S0025-5718-1987-0866109-5

[8] Debiao H, Jianhua C, and Jin H. An ID-based client authentication with key agreement protocol for mobile client-server environment on ECC with provable security. Information Fusion. 2012;13(3): 223–230. 10.1016/j.inffus.2011.01.001

[9] Chaum D, Rivest RL, and Sherman AT. Advances in cryptology. In Proceedings of CRYPTO. 1983;82: 279–303.

[10] Shieh WG, and Wang JM. Efficient remote mutual authentication and key agreement. computers and security. 2006;25(1): 72–77. 10.1016/j.cose.2005.09.008

[11] Lin CH, and Lai YY. A flexible biometrics remote user authentication scheme. Computer Standards and Interfaces. 2004;27(1): 19–23. 10.1016/j.csi.2004.03.003

[12] Hwang MS, Lee CC, and Tang YL. A simple remote user authentication scheme. Mathematical and Computer Modelling. 2002;36(1): 103–107. 10.1016/S0895-7177(02)00106-1

[13] Das ML, Saxena A, and Gulati VP. A dynamic ID-based remote user authentication scheme. Consumer Electronics. IEEE Transactions on. 2004;50(2): 629–631. 10.1109/TCE.2004.1309441

[14] Hwang MS, and Li LH. A new remote user authentication scheme using smart cards. IEEE Transactions on Consumer Electronics. 2000;46(1): 28–30. 10.1109/30.826377

[15] Yoon EJ, and Yoo KY. Robust id-based remote mutual authentication with key agreement scheme for mobile devices on ecc. In Computational Science and Engineering. CSE’09. International Conference on. 2009;2: 633–640.

[16] Yang JH, and Chang CC. An ID-based remote mutual authentication with key agreement scheme for mobile devices on elliptic curve cryptosystem. Computers and security. 2009;28(3): 138–143. 10.1016/j.cose.2008.11.008

[17] Islam SH, and Biswas GP. Comments on ID-based client authentication with key agreement protocol on ECC for mobile client-server environment In Advances in Computing and Communications. Springer Berlin Heidelberg 2011; 628–635.

[18] Zhang F, and Kim K. Efficient ID-based blind signature and proxy signature from bilinear pairings In Information Security and Privacy. Springer Berlin Heidelberg 2003; 312–323.

[19] Shim K. Efficient ID-based authenticated key agreement protocol based on Weil pairing. Electronics Letters. 2003;39(8): 653–654. 10.1049/el:20030448

[20] Paterson KG. ID-based signatures from pairingson elliptic curves. Electronics Letters. 2002;38(18): 1025–1026. 10.1049/el:20020682

[21] Nandakumar K. Multibiometric systems: Fusion strategies and template security. ProQuest. 2008.

[22] Wu M, Chen J, Zhu W, and Yuan Z. Security analysis and enhancements of a multi-factor biometric authentication scheme. International Journal of Electronic Security and Digital Forensics. 2016;8(4): 352–365. 10.1504/IJESDF.2016.079447

[23] Amin R, Islam SH, Biswas GP, Khan MK., and Li X. (2015). Cryptanalysis and enhancement of anonymity preserving remote user mutual authentication and session key agreement scheme for e-health care systems. Journal of medical systems, 39(11), 140 10.1007/s10916-015-0318-z 26342492

[24] Islam SK, Obaidat MS, and Amin R. An anonymous and provably secure authentication scheme for mobile user. International Journal of Communication Systems. 2016 10.1002/dac.3126

[25] Amin R, Kumar N, Biswas GP, Iqbal R, and Chang V. A light weight authentication protocol for IoT-enabled devices in distributed Cloud Computing environment. Future Generation Computer Systems. 2016 10.1016/j.future.2016.12.028

[26] Amin R, and Biswas GP. A secure light weight scheme for user authentication and key agreement in multi-gateway based wireless sensor networks. Ad Hoc Networks. 2016;36: 58–80. 10.1016/j.adhoc.2015.05.020

[27] Amin R, and Biswas GP. Design and analysis of bilinear pairing based mutual authentication and key agreement protocol usable in multi-server environment. Wireless Personal Communications. 2015;84(1): 439–462. 10.1007/s11277-015-2616-7

[28] Amin R, Islam SH, Biswas GP, Khan MK, Leng L, and Kumar N. Design of an anonymity-preserving three-factor authenticated key exchange protocol for wireless sensor networks. Computer Networks. 2016;101: 42–62. 10.1016/j.comnet.2016.01.006

[29] Amin R, and Biswas GP. Cryptanalysis and design of a three-party authenticated key exchange protocol using smart card. Arabian Journal for Science and Engineering. 2015;40(11): 3135–3149. 10.1007/s13369-015-1743-5

[30] Amin R. Cryptanalysis and Efficient Dynamic ID Based Remote User Authentication Scheme in Multi-server Environment Using Smart Card. IJ Network Security. 2016;18(1): 172–181.

[31] Li X, Niu JW, Ma J, Wang WD, and Liu CL. Cryptanalysis and improvement of a biometrics-based remote user authentication scheme using smart cards. Journal of Network and Computer Applications, 2011;34(1): 73–79. 10.1016/j.jnca.2010.09.003

[32] Li X, Niu J, Khan MK, and Liao J. An enhanced smart card based remote user password authentication scheme. Journal of Network and Computer Applications. 2013;36(5): 1365–1371. 10.1016/j.jnca.2013.02.034

[33] Li X, Niu J, Wang Z, and Chen C. Applying biometrics to design three factor remote user authentication scheme with key agreement. Security and Communication Networks. 2014;7(10): 1488–1497.

[34] Li X, Niu J, Khan MK, Liao J, and Zhao X. Robust three factor remote user authentication scheme with key agreement for multimedia systems. Security and Communication Networks. 2014 10.1002/sec.961

[35] He D, and Wang D. Robust biometrics-based authentication scheme for multiserver environment. IEEE Systems Journal. 2015;9(3): 816–823. 10.1109/JSYST.2014.2301517

[36] Ma CG, Wang D, and Zhao SD. Security flaws in two improved remote user authentication schemes using smart cards. International Journal of Communication Systems. 2014;27(10): 2215–2227. 10.1002/dac.2468

[37] Wang D, Ma CG, and Wu P. Secure password-based remote user authentication scheme with non-tamper resistant smart cards. In IFIP Annual Conference on Data and Applications Security and Privacy. Springer Berlin Heidelberg. 2012; 114–121.

[38] An Y. Security analysis and enhancements of an effective biometric-based remote user authentication scheme using smart cards. BioMed Research International. 2012. 10.1155/2012/519723 PMC3415263 22899887

[39] Das AK. Analysis and improvement on an efficient biometric-based remote user authentication scheme using smart cards. Information Security. IET. 2011;5(3): 145–151. 10.1049/iet-ifs.2010.0125

[40] Li CT, and Hwang MS. An efficient biometrics-based remote user authentication scheme using smart cards. Journal of Network and computer applications, 2010;33(1): 1–5. 10.1016/j.jnca.2009.08.001

[41] Al-Assam H, and Jassim SA. Multi-factor challenge/response approach for remote biometric authentication In SPIE Defense, Security, and Sensing. International Society for Optics and Photonics 2011; 80630V–80630V.

[42] Sarier ND. Improving the accuracy and storage cost in biometric remote authentication schemes. Journal of Network and Computer Applications. 2010;33(3): 268–274. 10.1016/j.jnca.2009.12.017

[43] Cox IJ, Miller ML, Bloom JA, and Honsinger C. Digital watermarking. San Francisco: Morgan Kaufmann 2002;53.

[44] Pointcheval D, and Zimmer S. Applied Cryptography and Network Security Proceedings. Springe: Berlin 2008;277–295.

[45] Choi Y, Lee D, Kim J, Jung J, Nam J, and Won D. Security enhanced user authentication protocol for wireless sensor networks using elliptic curves cryptography. Sensors. 2014;14(6): 10081–10106. 10.3390/s140610081 24919012 PMC4118368

[46] KAMAL K, GHANY A, MONEIM MA, GHALI NI, HASSANIEN AE, and HEFNY HA. A Symmetric Bio-Hash Function Based On Fingerprint Minutiae And Principal Curves Approach. 2011.

[47] Teoh AB, Goh A, and Ngo DC. Random multispace quantization as an analytic mechanism for biohashing of biometric and random identity inputs. Pattern Analysis and Machine Intelligence. IEEE Transactions on. 2006;28(12): 1892–1901. 10.1109/TPAMI.2006.250 17108365

[48] Kim J, Lee D, Jeon W, Lee Y, and Won D. Security analysis and improvements of two-factor mutual authentication with key agreement in wireless sensor networks. Sensors. 2014;14(4): 6443–6462. 10.3390/s140406443 24721764 PMC4029696

[49] Nam J, Choo KKR, Kim M, Paik J, and Won D. Dictionary attacks against password-based authenticated three-party key exchange protocols. KSII Transactions on Internet and Information Systems (TIIS). 2013;7(12): 3244–3260.

[50] Cao L, and Ge W. Analysis and improvement of a multi-factor biometric authentication scheme. Security and Communication Networks. 2015;8(4): 617–625. 10.1002/sec.1010

[51] Choi Y, Nam J, Lee Y, Jung S, and Won D. Cryptanalysis of Advanced Biometric-Based User Authentication Scheme for Wireless Sensor Networks In Computer Science and its Applications. Springer Berlin Heidelberg 2015;1367–1375.

[52] Nam J, Choo KKR, Kim M, Paik J, and Won D. An Offline Dictionary Attack against Abdalla and Pointcheval’s Key Exchange in the Password-Only Three-Party Setting. IEICE TRANSACTIONS on Fundamentals of Electronics, Communications and Computer Sciences. 2015;98(1): 424–427. 10.1587/transfun.E98.A.424

[53] Syverson P. A taxonomy of replay attacks [cryptographic protocols]. In Computer Security Foundations Workshop VII. CSFW 7. Proceedings. IEEE. 1994; 187–191.

[54] Otway D, and Rees O. Efficient and timely mutual authentication. ACM SIGOPS Operating Systems Review. 1987;21(1): 8–10. 10.1145/24592.24594

[55] Blake-Wilson S, Johnson D, and Menezes A. Key agreement protocols and their security analysis. Springer Berlin Heidelberg 1997; 30–45.

[56] Wen F, and Li X. An improved dynamic ID-based remote user authentication with key agreement scheme. Computers and Electrical Engineering. 2012;38(2): 381–387. 10.1016/j.compeleceng.2011.11.010

[57] Chou JS, Huang CH, Huang YS, and Chen Y. Efficient Two-Pass Anonymous Identity Authentication Using Smart Card. IACR Cryptology ePrint Archive. 2013;402.

[58] Das AK, and Goswami A. A secure and efficient uniqueness-and-anonymity-preserving remote user authentication scheme for connected health care. Journal of medical systems. 2013;37(3): 9948 10.1007/s10916-013-9948-1 23660745

[59] Boyd C, and Mao W. On a limitation of BAN logic In Advances in Cryptology-EUROCRYPT’93. Springer Berlin Heidelberg 1994;240–247.

[60] Bleeker A, and Meertens L. A semantics for BAN logic In Proceedings of the DIMACS Workshop on Design and Formal Verification of Security Protocols. 1997.

[61] Burrows M, Abadi M, and Needham RM. A logic of authentication In Proceedings of the Royal Society of London A: Mathematical. Physical and Engineering Sciences. The Royal Society 1989;426: 233–271.

[62] Abadi M, and Needham R. Prudent engineering practice for cryptographic protocols. IEEE transactions on Software Engineering. 1996;1: 6–15. 10.1109/32.481513

[63] Xue K, and Hong P. Security improvement on an anonymous key agreement protocol based on chaotic maps. Communications in Nonlinear Science and Numerical Simulation. 2012;17(7): 2969–2977. 10.1016/j.cnsns.2011.11.025

